# Metabotyping as a Stopover in Genome-to-Phenome Mapping

**DOI:** 10.1038/s41598-019-38483-0

**Published:** 2019-02-12

**Authors:** Pubudu P. Handakumbura, Bryan Stanfill, Albert Rivas-Ubach, Dan Fortin, John P. Vogel, Christer Jansson

**Affiliations:** 1 The Environmental Molecular Sciences Laboratory (EMSL), Pacific Northwest National Laboratory (PNNL), Washington, WA 99352 USA; 2 Advanced Computing, Computing and Analytics Division, PNNL, Richland, WA 99352 USA; 3 US Department of Energy (DOE) Joint Genome Institute (JGI), Walnut Creek, CA 94598 USA

## Abstract

Predicting phenotypic expression from genomic and environmental information is arguably the greatest challenge in today’s biology. Being able to survey genomic content, e.g., as single-nucleotide polymorphism data, within a diverse population and predict the phenotypes of external traits, represents the holy grail across genome-informed disciplines, from personal medicine and nutrition to plant breeding. In the present study, we propose a two-step procedure in bridging the genome to phenome gap where external phenotypes are viewed as emergent properties of internal phenotypes, such as molecular profiles, in interaction with the environment. Using biomass accumulation and shoot-root allometry as external traits in diverse genotypes of the model grass *Brachypodium distachyon*, we established correlative models between genotypes and metabolite profiles (metabotypes) as internal phenotypes, and between metabotypes and external phenotypes under two contrasting watering regimes. Our results demonstrate the potential for employing metabotypes as an integrator in predicting external phenotypes from genomic information.

## Introduction

As stated in the National Science Foundation’s (NSF) 2016 report, “10 Big Ideas for Future NSF Investment”^1^, The universally recognized biggest gap in our biological knowledge is our inability to predict the phenotype of a cell or organism from what we know about the genome and environment. This challenge to fully exploit the genome-to-phenome mapping potential has grown dramatically in recent years because of the speed and resolution by which we now can decipher genomic information through advanced high-throughput sequencing technologies. Most phenotypes are complex and quantitative in nature and a major quest in today’s life sciences lies in being able to use genomic and environmental information to predict multifaceted outcomes, be it human disease diagnostics and personal medicine, or animal and plant breeding.

**Figure 1:**
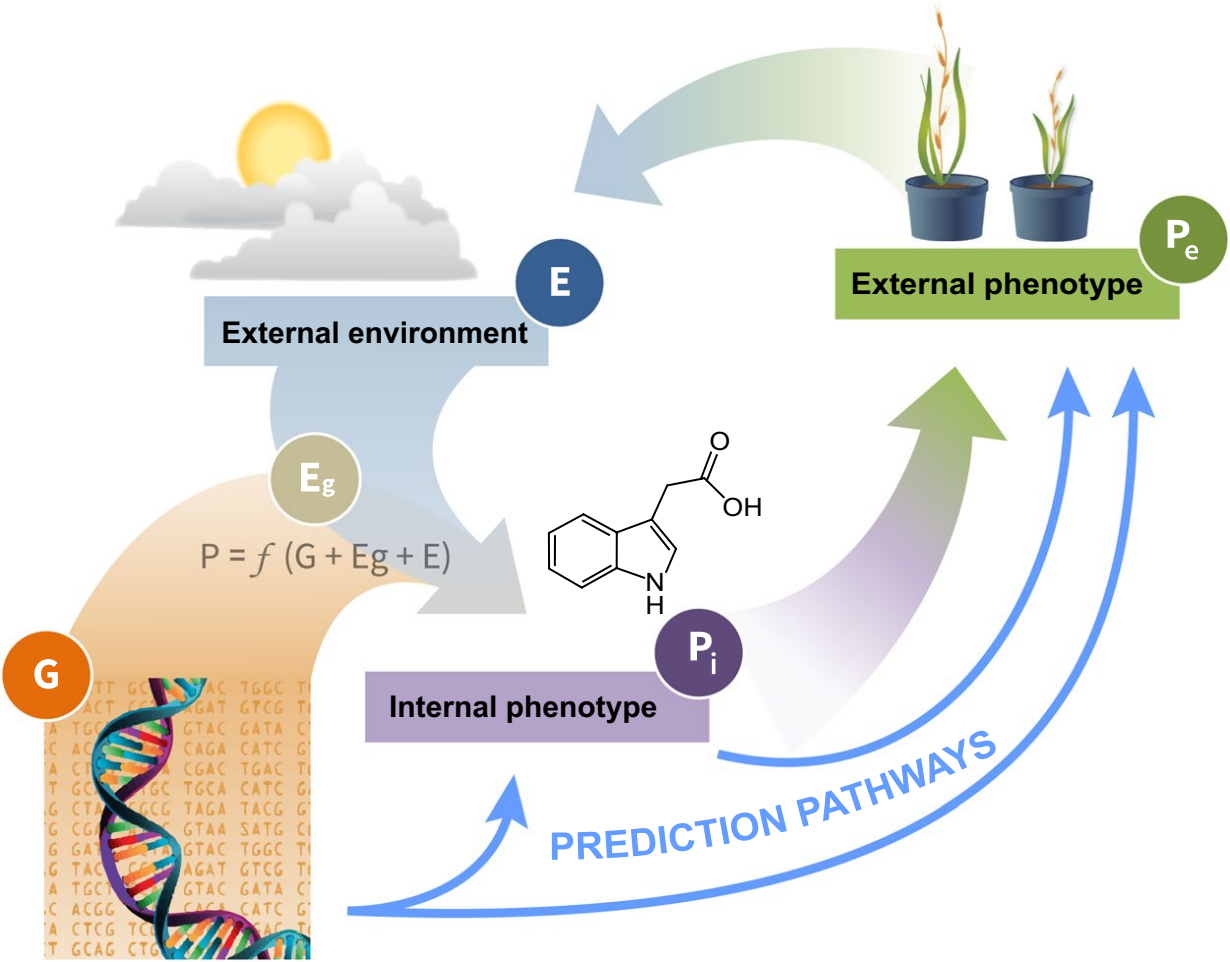
Genotype-phenotype predictions. The figure illustrates the relationships between plant genotype (G), plant epigenotype (E_g_), internal phenotype (P_i_; e.g., biochemical, physiological or cellular properties), external phenotype (P_e_; e.g., morphological or phenological properties), and the surrounding environment (E). Bridging the genotype-to-external phenotype gap is indicated by predicting external phenotype directly from genotypic information (e.g., from SNP genotyping), or via selected internal phenotypes (particularly molecular profiles) that can be clustered to genotypic information and associated with external phenotypes.

External phenotypes represent emergent properties, and as such are informed by internal phenotypes, e.g., biochemical and physiological properties, in interaction with the environment^2^. We argue that bridging the gap between genotype and external phenotypes can be facilitated by a two-step process, whereby linkages are established between genotype and internal phenotype on one hand, and between internal and external phenotypes on the other hand (Fig. 1). In considering internal phenotypes, we point to metabolomics as an evolving tool to provide insight into how genotypic diversity affects phenotypic variation in plants^3^. Although it should be noted, that the genetic control of plant metabolomes remains all but unknown^4^, and that even in a system such as *E*. *coli* interactions between gene variants and metabolite profiles are poorly understood^5^. A metabolome consists of thousands of low-weight compounds (metabolites) present in an organism at a specified moment^6^ and can be considered as the chemical phenotype (metabotype) of an organism^6^. Metabolites include products from cellular primary metabolism, such as sugars, nucleotides and amino acids, as well as from secondary metabolism, which are responsible for a large variety of complex physiological processes required to maintain cellular and organismal homeostasis and fitness. The metabolome is thus the final expression of a genotype, and is the first to respond to environmental perturbations^7^. Therefore, metabolites offer attractive attributes in phenotyping in that they reflect the integration of gene expression, protein interaction and upstream regulatory processes, and therefore can be considered as being closer to the phenotype of an organisms than the transcripts or proteins alone^8,9^. Finally, as opposed to proteomics, which requires a reference genome for meaningful data interpretation, metabolomics does not share that dependency as metabolites are not part of an organism’s coded information flow.

In the realm of plant biology, biomass accumulation, allocation of biomass between aboveground and belowground tissues (i.e., shoot-root allometry), and drought tolerance, represent complex quantitative yield-related plant functional traits (PFTs) that are critically important for our ability to select and/or design crops and cropping systems to meet increasing demand for plant biomass going into food, feed and energy production, while at the same time maintaining or increasing soil carbon. Functional, or optimal equilibrium theory holds that plants allocate resources among organs to optimize whole-plant fitness^10–12^. Thus, allocation of recent photosynthate between aboveground and belowground biomass for a given plant will shift in response to environmental variables such as soil moisture, light, and nutrient availability.

Shoot-root allocation of photosynthate is a complex quantitative yield-related PFT that can be described with the allometric equation^13,14^

1 $$y=b{x}^{k}$$

or its logarithmic conversion

2 $$\mathrm{ln}\,y=\,\mathrm{ln}\,b+k\,\mathrm{ln}\,x$$

where *y* is root biomass and *x* is shoot biomass, and *b* and *k* are constants with *k* being the allometric coefficient.

Bridging the genotype-to-phenotype gap relies on functional genome annotation of species with substantial genotypic diversity for targeted traits. As has been demonstrated for several plant species^15–19^, including *Brachypodium distachyon* (hereafter Brachypodium)^16^, allometric relationships may differ between plant genotypes. Exploring plant genotypic diversity for biomass accumulation and shoot-root allometry potentially enables selection of genotypes with high CO_2_ assimilation and specified allocation of photosynthate into aboveground and belowground biomass. For example, increases in root biomass with an extended root system architecture, offers a means to transfer more carbon to the soil as soil organic matter (SOM) through root exudation or via arbuscular mycorrhizal fungi, and allows more carbon to be incorporated into stabilized pools via physical or chemical sequestration, e.g., as soil aggregates or carbonate minerals, respectively^20^. Increased SOM, in turn, improves soil properties, rhizospheric microbiome community structure, nutrient use efficiency, water-use efficiency, crop yield, climate resiliency, and top soil erosion control^21,22^. In this context, it should be noted that allocation of resources between belowground and aboveground biomass is not necessarily a zero- sum game. Carbon allocation is a function of source-sink communication^23–25^ and there is ample evidence to suggest that plant photosynthesis is often feedback-inhibited by sink demand mediated via sugar signaling and its interaction with the environment^25–28^. Thus, within the constraints of available resources, increased investments in root biomass, with corresponding benefits in nutrient acquisition, water-use efficiency (WUE), and potential for long-term soil carbon storage do not always need to be at the expense of carbon allocation to harvestable portions of the plant. Rather it may result in a corresponding increase in source strength (i.e., photophosphorylation and carbon uptake and assimilation) to maintain adequate allocation of photosynthate to remaining sinks. This notion agrees with quantitative trait loci (QTL) analysis in poplar that demonstrated independent genetic control of aboveground and belowground biomass traits^29^.

Exploring genotypic diversity for PFTs such as biomass accumulation and allometry should allow development of plants and plant ecosystem for specified outcomes and biogeographical environments. As an example, it should be feasible to establish cropping systems with simultaneous high yield and extended root biomass and, hence, increased SOM and facilitated soil carbon storage. It is also conceivable to envision grasslands specifically developed to promote soil carbon storage, possibly combined with other ecosystem services such as nutrient cycling and trapping. A third example, on the other end of the spectrum, would be plants with decreased root biomass, exemplified by the transgenic low-methane, high-starch rice^30^. All these examples can be viewed within the larger context of ecosystem or rhizosphere engineering^31^.

Shifts in shoot-root allometry in response to changes in environmental conditions, e.g., increase in root: shoot ratio upon exposure to drought, are manifestations of phenotypic plasticity, i.e., the ability of plants to acclimate to altered conditions through regulatory networks. Genotypic diversity for plasticity responses offers opportunities to identify genotypes of plants that respond more favorably than others to environmental perturbations, e.g., plants that respond to drought by increasing allocation of photosynthate to belowground biomass while maintaining high yield of aboveground harvestable biomass.

Untargeted metabolomics has been successfully employed to establish relationships between metabolite profiles and quality traits in maize^9^, rice^32^ and potato^33^, biomass accumulation in sorghum^3^, drought tolerance in rice^34^, and growth rate in Arabidopsis^35^; (see^36^ for a review). Such metabotypes can be used as predictive molecular signatures for desirable traits in marker-assisted selection, which can significantly increase speed and reduce costs in breeding programs. Additionally, by unraveling molecular mechanisms underpinning desirable traits, metabotypes provide mechanistic understanding that can significantly facilitate further trait enhancements via crosses and/or synthetic biology.

This study is the first in a series of investigations where we seek to examine genotypic diversity for biomass traits in diverse accessions of the annual C_3_ grass *Brachypodium distachyon* (Brachypodium)^37^ across different environmental conditions, and if/how external and behavioral phenotypes can be predicted from metabolite profiles. The present study aims at exploring genotypic diversity for biomass accumulation and shoot-root allometry in Brachypodium under two contrasting watering regimes, well-watered and drought (henceforth referred to as control and drought conditions, respectively), and to what extent genotypic and external/behavioral phenotypic diversity correlate with metabolite profiles.

### Genotypic diversity for biomass accumulation and shoot-root allometry

**Figure 2:**
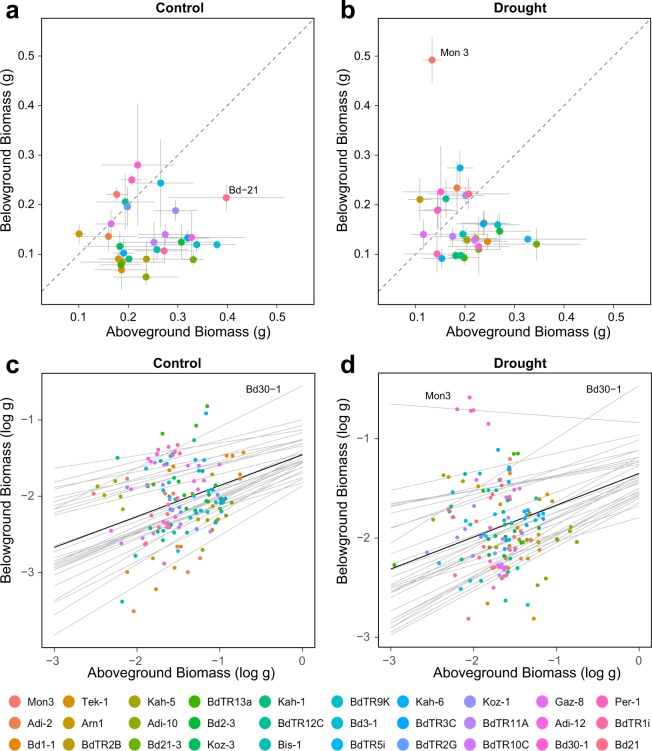
Genotypic diversity for aboveground and belowground biomass in 30 Brachypodium accessions under control (**a**,**c**) and drought (**b**,**d**) conditions. Individual points in each scatter plot (**a**,**b**) represent average values within accessions. The gray lines represent the uncertainty in the estimated mean (+/− standard error). The dashed line represents y = x; accessions below this line have greater aboveground biomass than belowground biomass. In the shoot-root allometry models (**c**,**d**) gray lines represent the estimated allometry relationship between aboveground and belowground biomass based on Eq. (2). Using a Bayesian hierarchical model. The allometric constant *k* is represented by the slope of the line for each accession. Individual points represent individual plants colored by accession. The solid black line represents the overall allometric relationship across accessions.

Biomass accumulation, measured as dry weight per plant for aboveground biomass (leaves + stems) and belowground biomass (roots), and shoot-root allometry varied substantially between genotypes under well-watered conditions (Fig. 2a,c, Extended Table 1). Total biomass accumulation ranged from 0.25 g to 0.7 g (2.8-fold change), aboveground biomass accumulation from 0.15 g to 0.45 g (3-fold change), and belowground biomass accumulation from 0.05 g to 0.25 g (5-fold change).

The genotypic diversity for root-shoot allometry based on equation(2) for the well-watered control group is shown in Fig. 2c. The allometric coefficient *k* is represented by the slope of the line corresponding to each accession where *k* is measured as posterior means for each accession using Bayesian hierarchical modelling. The value of *k* varied from 0.16 to 0.67. Shoot-root allometry, measured as root mass fraction (RMF, i.e. root biomass: total biomass) ranged from 0.17 to 0.68. Figure 2a,b versus c and d provide complimentary information on genotypic diversity by showing absolute and relative measures of plant biomass. Allometry curves for all accessions are presented in Extended Fig. 1.

Although, as expected, the general trend for drought-exposed Brachypodium plants during the recovery phase was a redirection of photosynthate from aboveground to belowground biomass, the effect of drought on biomass accumulation and allocation was considerably diverse among accessions, with lines exhibiting increase in aboveground biomass, belowground biomass and/or total biomass, albeit with different confidence band widths (Fig. 2b, Extended Fig. 2, Extended Table 1). Overall, the most notable diversity in drought response was found for belowground biomass, which varied from an increase of 123% to a decrease of 24%, and for total biomass accumulation with a range from an increase of 57% to a decrease of 36% (Fig. 2b, Extended Table 1). Perhaps the most striking response to drought was observed for Mon3 that showed statistically significant changes in both aboveground (decrease), belowground (increase), and total (increase) biomass (Fig. 2b, Extended Fig. 2, Extended Table 1). All the other accessions that showed increase in total biomass seemingly increased both aboveground and belowground biomass. Thus, Mon3 increased belowground biomass at the expense of aboveground biomass whereas the other accessions increased biomass belowground without a significant impact on the aboveground biomass, or deceased the aboveground biomass without a significant impact on the belowground biomass. Detailed statistical information for all data is given in Extended Table 1. Changes in shoot-root allometry were also visible as a result of the drought treatment. The most prominent change was again observed with Mon3. The effect of drought on Mon3 resulted in a change from a positive to negative *k* value in the allometry model, which distinguishes it compared to other accessions (Fig. 2d).

### Genotypic diversity for metabotypes

As is shown in Extended Table 2, the metabolomes for the 30 genotypes exhibited significant variation (Pseudo-F = 9.58; *P* < 0.0001), with a strong dependence on tissue type (Pseudo-F = 7.04; *P* < 0.0001) and water regime (Pseudo-F = 3.68; *P* < 0.0001). The largest metabolomic variance for all Brachypodium genotypes was found between tissue types (Pseudo-F = 389.98; *P* < 0.0001; see also Extended Fig. 3), which was also dependent on water regime (Pseudo-F = 13.73; *P* < 0.0001). Water regime alone had a significant effect on the metabolomic variance (Pseudo-F = 42.23; *P* < 0.0001).

**Figure 3:**
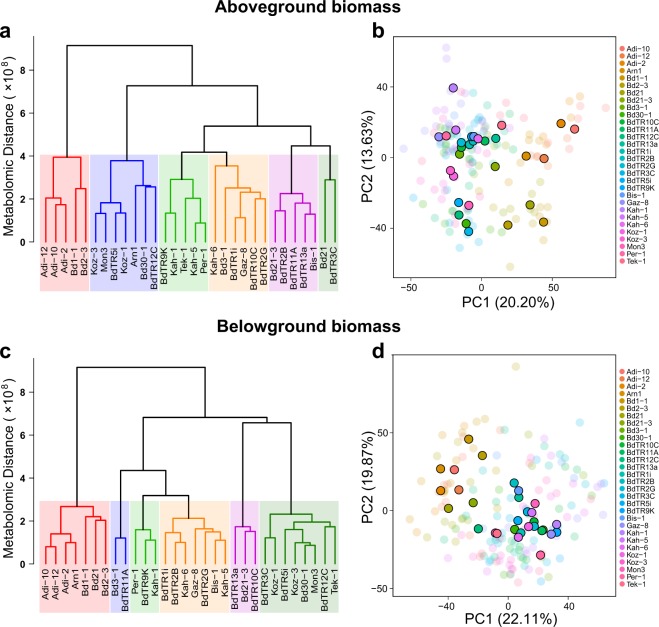
Cluster dendrograms and principal component analyses (PCAs) for the for aboveground and belowground biomass metabotypes of the 30 genotypes in the control group. Cluster dendrograms and PCAs for aboveground and belowground biomass were plotted separately. The six major clusters for aboveground (**a**) and belowground (**c**) biomass are indicated with different colors in the dendrograms. The metabolomic distance represented in the cluster dendrograms represents the Euclidian distance calculated between the averaged metabotype value for each genotype considering controls only. Different genotypes are designated with different colors in the case plots of the PCAs for aboveground (**b**) and belowground (**d**) biomass. Semi-transparent cases represent the biological replicates for each genotype, while solid colored cases represent their averaged values along PC1 vs. PC2 coordinates.

The metabolomic variance between genotypes (controls only) for aboveground and belowground biomass is illustrated through cluster dendrograms and principal component analyses (PCAs) in Fig. 3. We found that the genotypes Adi-12, Adi-10, Adi-2, Bd1-1 and Bd2–3 clustered together in dendrograms and presented the most distinct aboveground and belowground metabolomic profiles respect to the rest of genotypes (Fig. 3a,c). These findings were corroborated in PCAs for both aboveground and belowground biomass where those genotypes were separated from the rest along PC1. In addition, for belowground biomass, Arn1 and Bd21 also presented distant metabolic profiles that clustered with the above-mentioned genotypes (Fig. 3c). Baker’s gamma index for testing similarity between aboveground and belowground dendrograms was 0.62 and subsequent permutation test indicated that such similarity was statistically significant (P < 0.001). Therefore, the distances between the aboveground and belowground metabolic profiles for the different genotypes were statistically similar.

**Figure 4:**
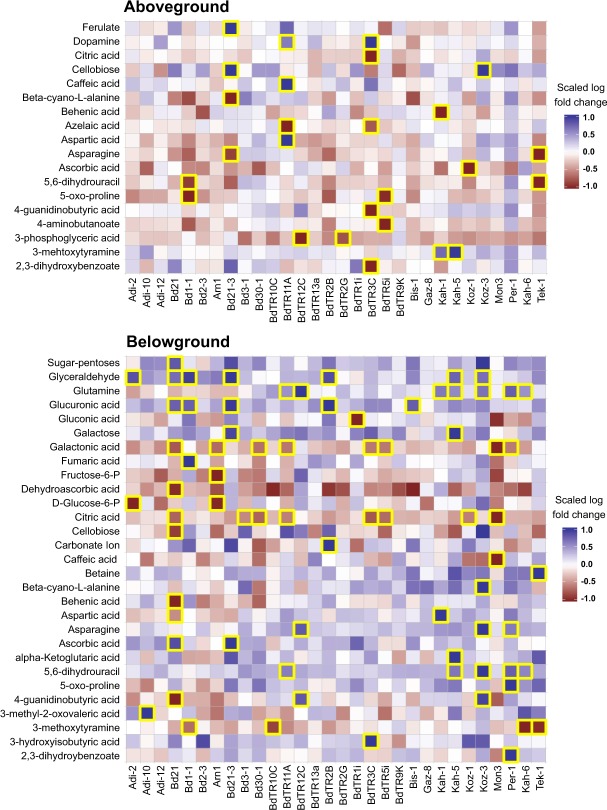
Metabolite heatmaps. Heatmaps representing the log-fold changes for known metabolites identified by GC- and LC-MS for aboveground (**a**) and belowground (**b**) biomass in response to drought treatment. Boxes with yellow edges indicate statistically significance at the 0.01 level after multiple comparisons. The log-fold changes have been standardized within each metabolite to highlight differences across the thirty accessions.

Holm corrected ANOVAs comparing plants under the two water regimes run on each of the 1,666 aboveground and 1,632 belowground metabolites revealed that the number of significantly changing metabolites varied between genotypes (Extended Fig. 4, Extended Tables 3 and 4). The highest number of significantly changed metabolites in aboveground biomass under drought conditions was found for Arn1, BdR11A, Bd21, BdTR3C, Bd1-1, whereas Koz1, BdTR1i, Gaz 8, BdTR2B and BdTR9K showed the least number of changed metabolites. The highest number of significantly changed metabolites for belowground biomass under drought was found for Bd21-3, Bd1-1, Arn1, BdTR2G, and Kah5, while Kah1, BdTR9K, BdTR10C, BdTR1i, while Gaz 8 showed the least number of changes. Identified metabolites exhibiting significant shifts in abundance between control and drought conditions across the 30 accessions is illustrated using heatmaps (Fig. 4). We found that belowground biomass had a higher number of significantly changed metabolites under drought conditions compared to aboveground biomass (Fig. 4). Proline, mannitol, galactose, tryptophan, hexose, phloroglucinol and purine showed a general increase in aboveground biomass levels in response to drought across genotypes. Levels of proline, galactose, betaine, glucuronic acid, phloroglucinol, hexoses and purine increased in root biomass across genotypes. In contrast, levels of ascorbic acid, 3-phosphoglyceric acid, ornithine, N-acetyl-D-glutamic acid, glutathione, glycine and asparagine decreased noticeably in aboveground biomass of drought-treated plants. 3-Methoxytyramine, phosphoric acid, citric acid, and uracil showed a decrease in root levels in response to drought across genotypes. Glyceraldehyde and glutamine were significantly enriched in belowground biomass in seven accessions (Adi-12, Bd21, Bd1-1, Bd21-3, BdTR2B, Kah-5 and Koz-3) while glucuronic acid and 5,6-dihydrouracil were significantly enriched in five accessions (Bd21, Bd1-1, Bd21-3, BdTR2B and Bis-1 and BdTR11A, Kah-5, Koz-3, Per1 and Kah-6 respectively). On the other hand, galactonic acid and citric acid abundance was significantly decreased in eight accessions (Bd21, Arn1, Bd30-1, BdTR11A, BdTR3C, BdTR5i, Mon3, and Per1 and Bd21, Bd3-1, Bd30-1, BdTR11A, BdTR3C, BdTR5i, Koz-1 and Mon3 respectively).

### Building genotype-metabotype maps

**Figure 5:**
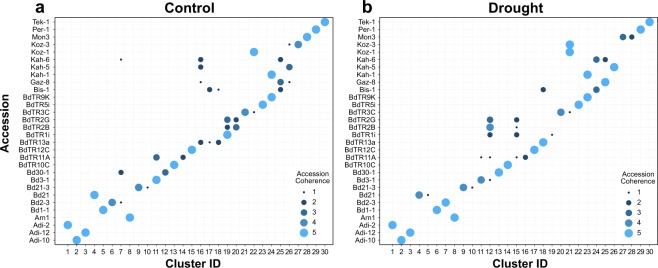
Bubble plots of genotype-metabolite associations. Association between the 30 Brachypodium genotypes and metabotypes for (**a**) control and (**b**) drought conditions. Thirty clusters were created using hierarchical clustering of metabolites measured by GC- and LC-MS for aboveground and belowground tissues for each plant. The circles on the plot indicate how many plants of a given genotype reside in each cluster—we consider this number to be a measure of accession coherence with respect to the metabotype.

Having demonstrated genotypic diversity for external traits (*i*.*e*., biomass accumulation and shoot-root allometry) and internal traits (*i*.*e*., metabolite profiles) in the Brachypodium accession panel, we wanted to find out to what extent the observed genotypic diversity for metabolite profiles (Extended Fig. 5a,b) could be clustered to reflect genotype-metabotype linkages. To probe this question, we used hierarchical clustering to group the metabotypes into 30 clusters based solely on relative abundance of all metabolite features (Extended Tables 3 and 4) and asked if the five individuals for a given genotype could be mapped to a single metabotype cluster. The results are illustrated in a bubble plot (Fig. 5) as it allowed us to represent genotype and metabotype clusters together with a third variable depicting the level of agreement between the metabolite and genotype clusters. The charts in Fig. 5 show that 17 genotypes (57%) in the control group and 13 genotypes (43%) in the drought group, respectively, could be linked to a unique metabotype cluster. Thus, for plants in the control group, a majority of the genotypes have metabolite profiles unique enough to statistically distinguish them from the other genotypes; a result similar to what was found by comparing the metabotype dendrograms in Fig. 3a,b via Baker’s gamma index. The lower value for the drought group implies that the different genotypes use similar metabolic mechanisms to cope with the drought stress, which drives their metabotypes to look similar across genotypes. Extended Table 5 shows the number of metabotype clusters contained in a given genotype.

### Mapping metabotypes to external phenotypes

**Figure 6:**
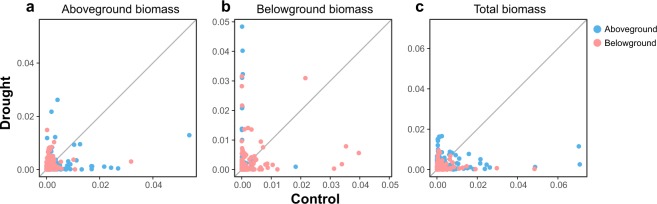
Random Forest prediction of significant metabolites. Random Forest model uses genotype identifiers and metabotypes to predict aboveground (**a**), belowground (**b**) and total biomass (**c**) changes under control and drought conditions. The importance of each metabolite for control and drought conditions after controlling for genotypic effects are plotted with aboveground and belowground metabolites in blue and pink, respectively. Relatively large importance values imply that a metabolite is more important than others to predicting changes in biomass.

Several statistical models, including linear models, non-linear regression, artificial neural nets, and Random Forest (RF) models were investigated as potential mapping tools to link metabotypes to aboveground and belowground dw biomass data. A RF model was chosen as it offers a good predictive power and provides the ability to identify particularly predictive metabolites (Fig. 6). Moreover, this model allows the comparison between drought and control conditions.

The noticeable differences in biomass traits across genotypes is being accounted for in the RF model by the genotype identifiers. Therefore, the model attempts to identify metabolites that affect biomass beyond what is explained by genotypic effects. Variable importance metrics derived from the fitted model were used to highlight influential metabolites in the model (Fig. 6, Extended Fig. 6). For describing aboveground biomass under control conditions most of the important metabolites are found in the aboveground biomass metabolome, though some belowground metabolites are also indicated as important. The root mean squared error (RMSE) for this model is 0.06 (Extended Table 6), which can be interpreted to mean that the average error in predicted aboveground biomass is 0.06 g. Extended Table 7 shows a list of the top-20 metabolites explaining the biomass under drought and/or control conditions in order of importance. Another notable observation is that the aboveground metabolite profile significantly contributed to explaining the change in biomass under drought and control conditions. This can be quantified by observing the increase in RMSE when predicting aboveground biomass using only aboveground metabolites. In the control group, the RMSE increased by 11% and in the drought group by 20% (Extended Table 6). Thus, the accuracy of the model decreases in both control and drought groups when root metabolites are not included in the model, with the largest drop occurring in the drought group. This is in agreement with the bubble plots where we observe a larger variability in root metabolism.

## Conclusion

**Figure 7:**
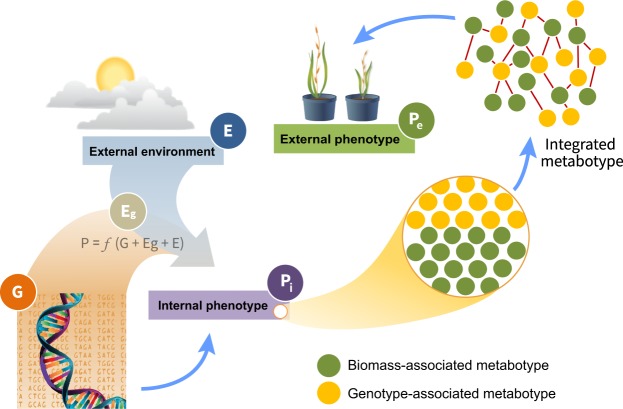
Proposed genotype-to-phenotype mapping via metabotypes. In this case, the internal phenotype contains two sets of metabotypes, one generated by linkage to genotypes and one by linkage to biomass traits. Integrated analysis that searches for correlated signatures between the independently derived metabotypes should aid in predicting biomass phenotypes for a given genotype and, by extension, from genomic sequence information.

One major quest in plant functional genomics is to connect genotype to phenotype and use this information to make phenotypic predictions and select superior genotypes for continued improvement efforts. We propose that metabotyping offers the potential to significantly facilitate the translation of genomic information to phenotypic expression of external traits. In this study, we used correlative modeling with a relatively small number of specimens to assign genotype-specific metabotypes and to link metabotypes to biomass traits under two different environmental conditions. We envision, that with an appropriately large cohort and number of biological replicates, and with continued development of data-driven methods to advance computational signal discovery algorithms, the strategy described here should allow for linking genomic information to external phenotypes via deconvolution of complex metabolite data sets (Fig. 7).

## Methods

### Plant material and growth conditions

Thirty diverse lines from the 54 re-sequenced *Brachypodium distachyon* (Brachypodium) genotypes in the Brachypodium pan-genome project, BrachyPan (https://brachypan.jgi.doe.gov) at the Department of Energy (DOE) Joint Genome Institute (JGI; Walnut Creek, CA, USA) were selected^38^. Seed availability and geographic locations were also factored into the selection^39,40^. Brachypodium accessions were obtained from the JGI Brachypodium germplasm collection (https://jgi.doe.gov/our-science/science-programs/plant-genomics/brachypodium/). The Brachypodium genotypes used in this study represent ecotypes originally collected from Turkey, Spain and Iraq (Extended Fig. 7). Details are provided in Extended Table 8). Plants were grown in phytotron under two contrasting water regimes, well-watered and drought spell, henceforth referred to as control and drought conditions, respectively.

To synchronize germination, seeds were imbibed in water in moist paper towels at 22 °C for three days before planting. Individual seeds were planted on 3″ pots containing 60 g of MVP profession growing mix (Hummert international) and grown in a controlled environment phytotron at 22 °C during day and 18 °C at night with 16:8 h light: dark cycles. An ambient CO_2_ concentration (∼400 ppm), relative humidity of 60% and a light intensity 350 μmol m^−2^ s^−1^ was used throughout the growth period. Twenty seeds from thirty different lines were grown and tested.

Twenty Brachypodium individuals of each genotype were grown in two different water regimes: 1) by adding 50 ml of water every other day from start to end (control), and 2) by adding 50 ml of water every other day until the 22^nd^ day after planting (plant were at tillering stage, few days prioir to booting) following a 6-day pre-flowering drought treatment by withholding water (drought). At the end of the drought treatment soil moisture was recorded as the volumetric water content (VWC). This a numeric value presented as the ratio of the volume of water for the volume of soil used. At the end of the 6^th^ day of drought treatment the average VWC for control group was 31.6 and the average VWC for drought group was 4.2. (Extended Fig. 8). Control and drought-treated plants were re-watered once after the 6-day drought period and allowed to recover for 24 h before harvesting. All plants were grown for a total of four weeks from seed sowing to harvest and samples were collected just before flowering.

### Phenotypic data collection, tissue harvest and sample processing

Aboveground (leaves + stems) and belowground (roots) fresh weight were manually recorded for ten replicates of each accession. Immediately after phenotypic data collection, the aboveground portion of each plant was harvested, weighed, and flash frozen in liquid nitrogen. Roots from each plant were washed and dried with paper towels, weighed and immediately flash frozen in liquid nitrogen. Of the ten flash-frozen replicates from each treatment and accession five were used for dry biomass measurements following lyophilization and five were saved for proteomics analysis. The lyophilized samples were ground using a Qiagen TissueLyzer II (Germantown, MD, USA) and sample powders were kept at −80 °C until metabolite extraction.

### Metabolite extraction for mass spectrometry (MS) analyses

Polar and semi-polar metabolites were extracted as described elsewhere^41^ but with minor modifications. Briefly, for each sample, 40 mg of lyophilized powder were added into a clean 2 mL glass vial. Subsequently, each glass vial received 1 ml of methanol: water (80:20). Samples were shaken in a Thermomixer (Eppendorf, Hamburg, Germany) at 1,200 rpm for 1 hour at 21 °C and subsequently centrifuged at 13,000 × g for 10 min. Supernatants were collected and split into two clean HPLC vial sets; 300 μL for gas chromatography MS (GC-MS) and other 300 μL for liquid chromatography MS (LC-MS) analyses.

Supernatants for GC-MS analyses were dried completely in a vacuum evaporator and extracts were derivatized to trimethylsilyl esters^42^. For derivatization, each vial received 20 µL of methoxyamine in pyridine solution (30 mg/mL) and samples were incubated in a Thermomixer (Eppendorf, Hamburg, Germany) at 1,200 rpm for 90 min at 37 °C. After the first incubation, each vial received 80 μL of MSTFA (N-Methyl-N-(trimethylsilyl) trifluoroacetamide) and incubated at 1,200 rpm for 30 min at 37 °C to derivatize amine, carboxyl and hydroxyl groups. After incubations, vials were vortexed for 10 seconds and centrifuged for 1 min at 13,000 × g. Supernatants were transferred with Pasteur pipettes into clean glass HPLC vials with 200 µL inserts.

### GC-MS analyses

Derivatized samples were analyzed by an Agilent GC 7890A coupled to a MSD 5975C mass spectrometer (Agilent Technologies, Santa Clara, CA). Samples were randomized prior to injections. A HP-5MS column (30 m × 0.25 mm × 0.25 μm; Agilent Technologies) was used for the GC. The injection volume was set at 10 µL and split-less. The injection port temperature was maintained at 250 °C. The column oven was maintained for 1 min at 60 °C and then temperature increased to 325 °C at a rate of 10 °C/min (26.5 min ramp) and hold for another 10 min. A mixture of fatty acid methyl esters (FAMEs; C8-C28) was analyzed at the beginning of the each day sequence and an experimental blank was injected every 15 samples. Peak area of the fatty acid methyl mystate (C14; 15, 57 min RT; 1,400 Retention Index) was used for sample scaling purposes. Instrument sensitivity experienced <15% of fluctuation between sequences. Experimental blanks, consisting in derivatized dried methanol: water (80:20), were used for instrument background filtering.

### Processing of GC-MS chromatograms

GC-MS raw files were processed with Metabolite Detector 2.5^43^. Before processing, “Agilent.D” files were converted to netCDF format with Agilent Chemstation and posteriorly converted to “bin” files directly with Metabolite Detector 2.5 software. GC-MS chromatograms were deconvoluted, aligned and metabolites were identified before exporting the dataset to a CSV file format. Briefly, the analysis of the FAMEs mixture allowed the calculation of the retention indices (RI) for each detected metabolite. Chromatograms were then aligned and deconvoluted. First metabolite identification was performed by matching MS spectra and RI to an updated in-house version of FiehnLib^44^ which contains over 850 metabolites with validated spectra and RIs. Probability threshold for metabolite matching was set at 70%. Assigned metabolites were subsequently verified by matching fragmented spectra from NIST14 GC-MS library. Parameters used in metabolite detection are shown in Extended Table 9. Metabolite matching information in GC-MS is shown in Extended Table 10. For more details of metabolite assignation with GC-MS see^45^.

### LC-MS analyses

A high-resolution LTQ Orbitrap Velos mass spectrometer (HRMS) with a heated electrospray ionization (HESI) source (Thermo Fisher Scientific, Waltham, Massachusetts, USA) coupled to a Vanquish ultra-high pressure liquid chromatography (UHPLC) system (Thermo Fisher Scientific, Waltham, Massachusetts, USA) was used to obtain the LC-MS chromatograms. LC was performed using a C18 Hypersil gold reversed-phase column (150 × 2.1 mm, 3 µm particle size; Thermo Scientific, Waltham, Massachusetts, USA). The column compartment of the UHPLC system was maintained at 30 °C. Mobile phases consisted in 0.1% formic acid in water (A) and acetonitrile/0.1% formic acid in water (90:10) (B) and were filtered and degassed for 20 min in an ultrasound bath before use. The injection volume was set at 10 µL. Flow rate was maintained constant at 0.3 mL min^−1^ during chromatography. The elution gradient initiated at 90% A (10% B) and was maintained for 5 min, then the gradient ramped linearly to 10% A (90% B) during the next 15 min. Those conditions were held for 2 more min and the initial proportions (90% A; 10% B) were thus linearly recovered over the next 2 min. The column was washed and stabilized for 11 more minutes at the initial conditions. All samples were analyzed in in negative (−) ionization mode. Samples were randomized prior to injections. The HRMS operated in FTMS (Fourier Transform Mass Spectrometry) full-scan mode at a resolution of 60,000 and a mass range of 50-1000 m/z. Mass accuracy and sensitivity of the instrument was monitored by injecting a mixture of standards (caffeic acid, folic acid and quercetin) at frequent intervals during the sequence. The HRMS was calibrated to <1ppm error every 3 days of operation. A Daidzein (253.0506 m/z (-H); 11.9 min RT) standard at 30 mM was injected daily during the sequence for sample scaling purposes to cope with instrument sensitivity variability. Instrument sensitivity fluctuated <10% between sequences. Experimental blank samples, consisting of methanol: water (80:20), were injected every 15 samples and used for instrument background filtering. See^46^ for a more detailed HRMS operation instructions.

### Processing of LC-MS chromatograms

The HRMS RAW files were processed by MZmine 2.26^47^. Chromatograms were baseline corrected, deconvoluted, aligned and metabolites were identified before exporting the dataset to a CSV format file. The parameters used for the extraction of the metabolic fingerprints are given in Extended Table 11. Metabolites in LC-MS chromatograms were assigned based on the exact mass and the retention time (RT) of the deconvoluted ions from chromatograms and standards. Our standard library includes over 500 common metabolites from primary and secondary metabolism typically present in plants. Although our LC-MS metabolite assignment is considered putative^48^, the high mass accuracy achieved by HRMS coupled to highly reproducible RT substantially decreases the number of false positive assignations. For more details of metabolite assignation see^49^. RT and m/z values for LC-MS metabolite identification are shown in Extended Table 12.

### Metabolomics data filtering

GC-MS and LC-MS datasets were filtered before statistical analyses through four main steps: 1) background signal obtained from the experimental blank samples were removed from all samples; 2) Zero values were replaced for missing data (NAs); 3) Variables with data present in less than three individuals in all cell factors (Genotype × Water regime × Plant tissue) were removed from the data set. 4) Outlier values for each cell factor and variable were replaced for NAs and were detected as follows:

$$Upper\,outliers\,\to value > Q3+3\times IQR$$

$$Lower\,outliers\to value < Q1-3\times IQR$$

where Q3 is the 3^rd^ quartile (75^th^ percentile), Q1 is the 4^th^ quartile (25^th^ percentile) and IQR is the interquartile range (IQR = Q3-Q1).

### Statistical analysis

The generated dataset for this study included three different categorical factors: “Genotype” with 30 levels (Adi-2, Adi-10, Adi-12, Bd21, Bd1-1, Bd2-3, Arn1, Bd21-3, Bd3-1, Bd30-1, BdTR10C, BdTR11A, BdTR12C, BdTR13a, BdTR2B, BdTR2G, BdTR1i, BdTR3C, BdTR5i, BdTR9K, Bis-1, Gaz-8, Kah-1, Kah-5, Koz-1, Koz-3, Mon3, Per1, Kah-6, Tek-1), “Plant tissue” with two levels (aboveground and belowground biomass), and “Water regime” with two levels (control and drought). The dataset was composed of a total of 2,898 continuous variables: biomass measurements and 2,897 metabolite features detected with LC-MS and GC-MS. A total of 126 variables were identified and the remaining 2,771 variables remained as unknown metabolite features (unknowns).

A linear model that allows for different variances for each accession was used to determine if the biomass in the drought group was significantly different from the control group. This type of model is often referred to as an Aitken model.

The logarithmic conversion of the allometric model (equation 2) was fit using a Bayesian hierarchical model (BHM). A BHM was used instead of a standard regression fit to each accession because we hypothesized that the allometric equations should be similar for all accessions of the same species, i.e. *Brachypodium distachyon*. Therefore, individual regression fit to each accession would ignore the common, genotypic information shared across accessions while a single regression fit to all accessions would ignore the different responses to drought that differentiate the accessions. The fitted BHM allows each accession to have a unique allometric model which sharing information across accessions due to genotypic correlations.

The overall metabolomic changes between the levels of the studied factors and their interactions were analyzed through a full-factorial permutational multivariate analysis of variance (PERMANOVA) using the Euclidian distance (Metabolomic variation = Genotype + Plant tissue + Water regime + Genotype × Plant tissue + Genotype × Water regime + Plant tissue × Water regime + Genotype × Plant tissue × Water regime)^50^. For this PERMANOVA, the entire metabolomics dataset including the 2,897 metabolite features from LC-MS and GC-MS was used. The number of permutations was set to 10,000.

To determine which metabolites were differentially abundant in the drought group versus the control for each accession, the raw abundance values were transformed to log base 2, then metabolites were filtered using the ANOVA filter^50^. That is, due to the fact that at least two data points are required to estimate the within treatment-by-accession variance, all metabolites that appeared less than twice in every treatment-by-accession combination were removed from analysis. Of the 1,990 identified metabolites, 79 and 52 metabolites were removed from the root and aboveground biomass analyses, respectively. All of the remaining metabolites were analyzed using an ANOVA test with a significance level of 0.01. The Holm adjustment to correct *P* values for multiple comparisons^50,51^ was used to identify which metabolites were differentially abundant in the two groups within each genotype.

Additionally, aboveground and belowground data from the entire metabolomics fingerprints for the control plants of the 30 genotypes were separately submitted to dendrogram analysis and to Principal Component Analysis (PCA) to explore the natural variability between Brachypodium genotypes. The dendrograms were created via hierarchical clustering based on the squared Euclidean distance between the plant metabolite profiles. The plants were clustered using a variation of the Ward’s method where the dissimilarities between the clusters are squared before cluster updating^52^. The number of clusters was then reduced from 150 (one for each plant) to 30 to parallel the number of genotypes present in the data. The resultant dendrograms were compared using Baker’s gamma index, which measures the association between dendrograms generated by hierarchical clustering^53^. To determine the statistical significance of the computed index value, the labels on the dendrogram nodes were permuted and the index was calculated again. This was repeated 1000 times to approximate the null distribution of the gamma conditioned on the observed tree structures. Because none of the permuted gamma indices were greater than the observed value, 0.62, the trees were determined to be statistically similar.

In order to cope with any effects of collinearity between metabolites and the complex relationship between metabolite abundance and biomass measurements, a variety of modelling approaches were used to quantify the correlation between metabolite abundances and biomass measurements. Two forms of regularized regression (ridge regression and LASSO) were fit to the data, but the linearity assumption of these models proved too rigid to model the input/output relationship adequately. To address this shortcoming, non-linear machine learning methods (neural networks and support vector machines) were fit to the data, but these methods made it difficult to identify exactly which metabolites were most highly correlated with biomass and were therefore not used in the final analysis. Random forests were used because they are sufficiently flexible to capture the complicated input/output relationship and are transparent enough to identify which metabolites are most predictive of biomass. The random forest models were fit using five-fold cross-validation to tune a final random forest model fit to the full dataset. The prediction statistics presented in the appendix are those derived by averaging the prediction statistics computed during the five-fold cross validation phase.

All statistical analyses were performed in R version 3.4.1^54^ and the source code is available for download from the GitHub repository “PREMIS-metabotyping” at the website https://github.com/PNNL-PREMIS/PREMIS-metabotyping. The BHM model was fit using the programming language Stan^55^. Metabolite analysis was done using the *pmartR* library in R (https://github.com/pmartR). PERMANOVAs were conducted with the *adonis* function in “vegan” package^56^. Cluster dendrograms were plotted using the *hclust* function in “stats” package^54^. For the PCA, the missing data were imputed with *imputePCA* function from “missMDA” package^57^. After this step, PCA was calculated with the *PCA* function from “FactoMineR” package^58^. The parameter SCALE from the *PCA* function was set at TRUE for scaling the matrix before the analysis. The random forest analysis was accomplished using the *caret* package, version 6.0–76^59^. Baker’s gamma index was computed using the “dendextend” package^60^.

## Supplementary information

Supplementary Information

## Data Availability

Interactive heatmaps and associated data sets are available at https://ascm.shinyapps.io/BAS_gobrachy/. Source code is available for download from the GitHub repository “PREMIS-metabotyping” at the website https://github.com/PNNL-PREMIS/PREMIS-metabotyping.
