# Supplementary material for: Metabotyping as a Stopover in Genome-to-Phenome Mapping
Source: Sci Rep. 2019 Feb 12;9:1858. doi: 10.1038/s41598-019-38483-0 (PMC6372633; doi:10.1038/s41598-019-38483-0)
Supplement: Supplementary file 1 — Supplementary Information [file 41598_2019_38483_MOESM1_ESM.pdf]

Supplementary Information

Metabotyping as a Stopover in Genome-to-Phenome Mapping

Pubudu, P. Handakumbura[1], Bryan Stanfill[2], Albert Rivas-Ubach[1], Dan Fortin[2], John Vogel[3], Christer Jansson[1]

[1]The Environmental Molecular Sciences Laboratory (EMSL), Pacific Northwest National Laboratory (PNNL), WA 99352
[2]Advanced Computing, Computing and Analytics Division, PNNL, Richland, WA 99352
[3]US Department of Energy (DOE) Joint Genome Institute (JGI), Walnut Creek, CA 94598

Metabotyping as a Stopover in Genome-to-Phenome Mapping

Pubudu, P. Handakumbura[1], Bryan Stanfill[2], Albert Rivas-Ubach[1], Dan Fortin[2], John Vogel[3], Christer Jansson[1]

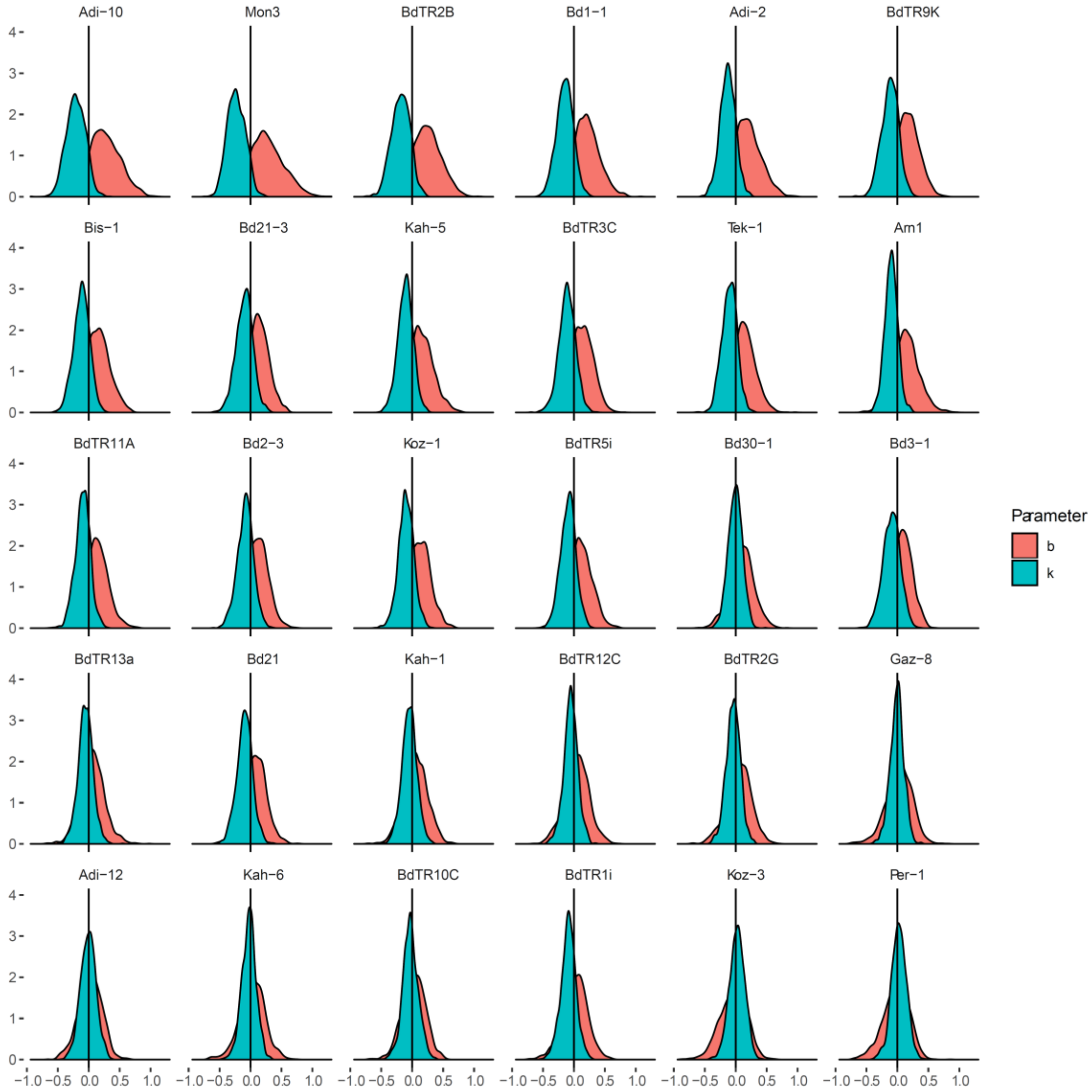

Supplementary Data Fig. 1. Posterior distributions of the difference in parameters of the allometric model (equation (1)) between the control and drought condition for each accession based on the hierarchical Bayesian model. The further the distribution is away from zero (marked by the vertical line) the more evidence for a change in the allometric parameter between the control and drought conditions.

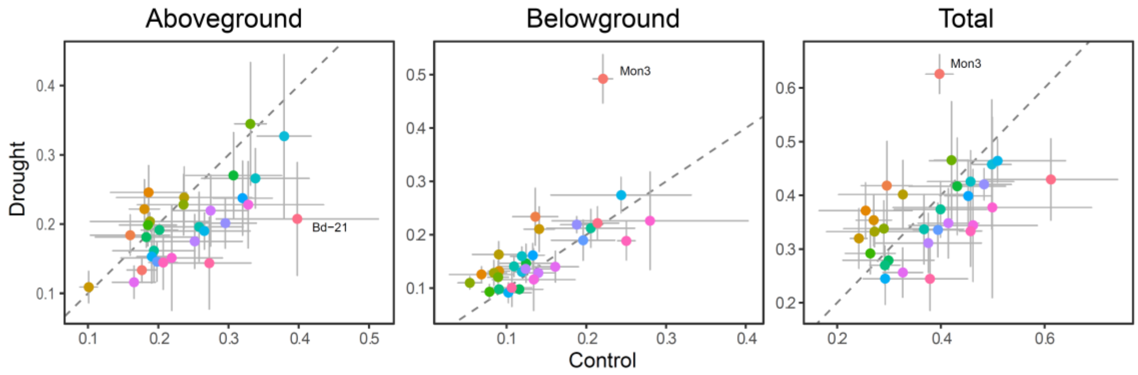

Supplementary Data Fig. 2. Scatter plots showing aboveground (left), belowground (center) and total biomass (right) of 30 Brachypodium accessions under control and drought conditions. Individual points represent average values within accessions. The gray lines represent the uncertainty in the estimated mean (+/- standard error). The dashed line is the line y = x, accessions below this line have greater biomass under control conditions and accessions above this line have a greater biomass under drought conditions.

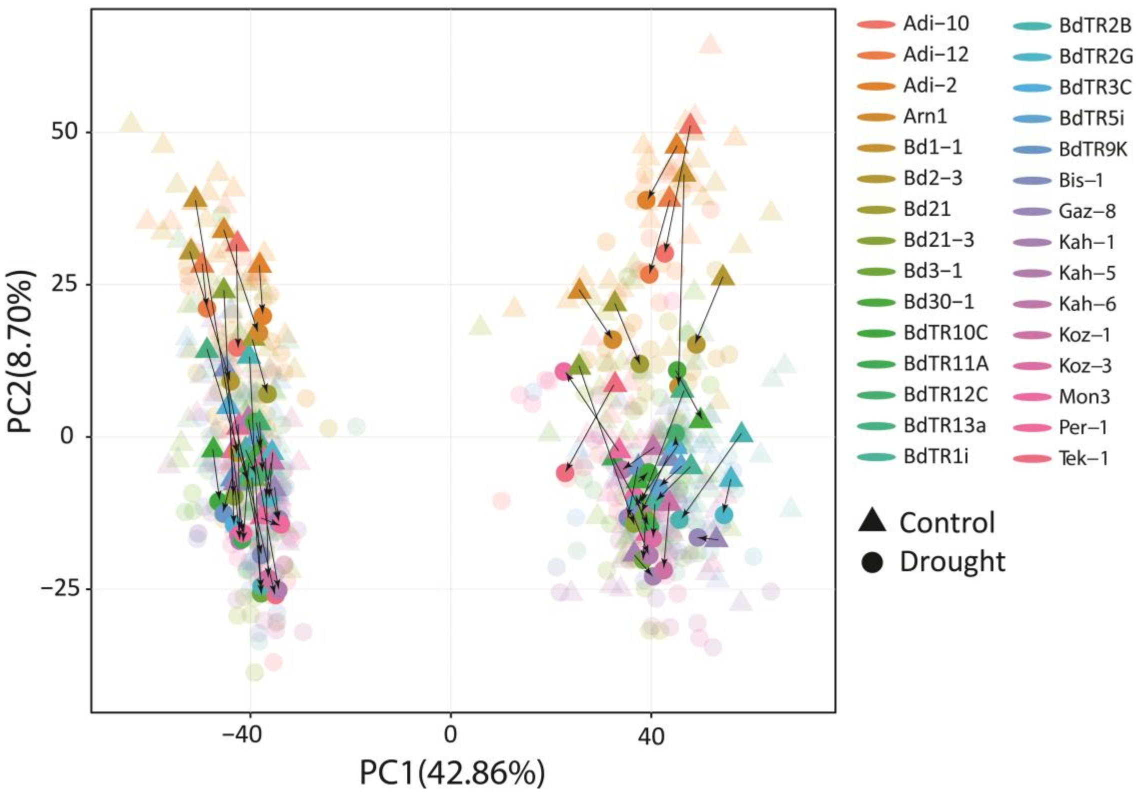

Supplementary Data Fig. 3. Principal component (PC) 1 vs. PC2 of the case plot of the PCA performed with the metabolomics dataset including plant tissues (aboveground and belowground biomass), treatments (control and drought conditions), and the 30 genotypes. Different genotypes are represented with different colors. Plants grown under control and drought conditions are indicated by triangles and circles, respectively. Cases represented in negative values along the PC1 correspond to belowground biomass and cases represented in the positive values along the PC1 correspond to aboveground biomass. Semi-transparent cases represent the biological replicates of the metabolomics dataset, while solid colored cases represent their averaged values along PC1 vs. PC2 coordinates. Black arrows indicate the direction of the metabolomic change from control to drought conditions for each genotype in the PC1 vs. PC2 plane for aboveground and belowground biomass.

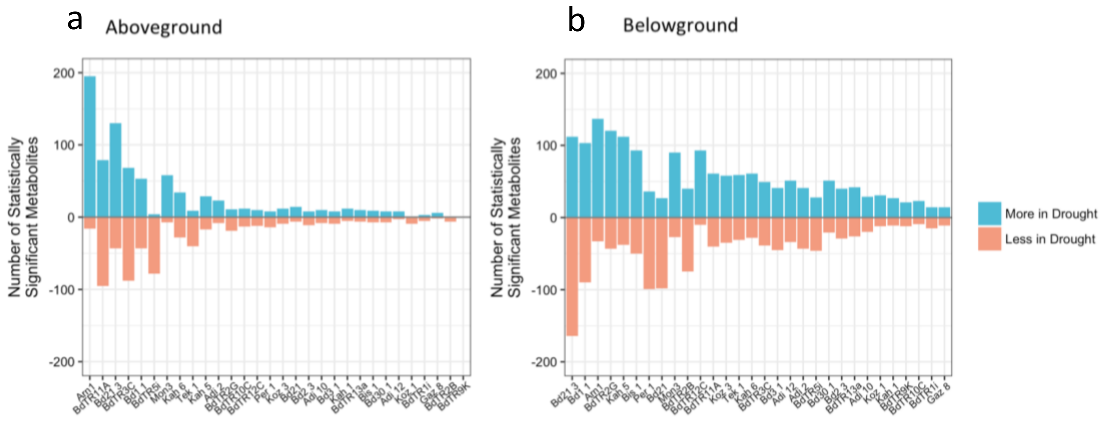

Supplementary Data Fig. 4. The total number of differentially abundant metabolites in aboveground (A) and belowground (B) biomass in the control and drought groups that are statistically significant at the 0.01 level after accounting for multiple comparisons. The blue bars represent the number of metabolites that are more abundant in the drought group relative to the control, and vice-versa for the maroon bars. A total of 2897 metabolic features were considered in the above analysis.

a

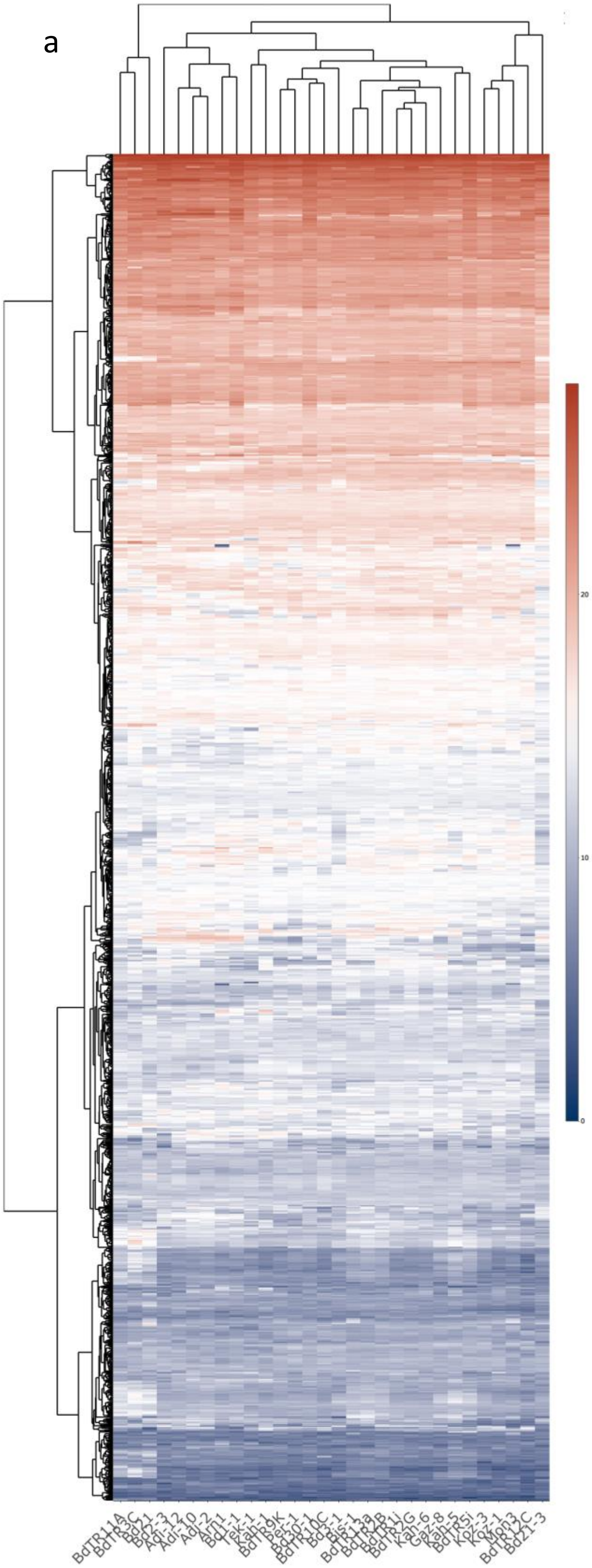

Supplementary Data Fig. 5a. Metabolomic diversity of the 30 Brachypodium genotypes. The average log 2 abundances for each metabolite and accession exposed to the control treatment are plotted in two heatmaps, one for each tissue, aboveground (A) and belowground (B) biomass, where the colors indicate the abundance of each metabolite. The metabolites and genotypes were clustered hierarchically using Ward's method. Genotypes were clustered based on the Euclidean distance between metabolite profiles in the respective groups and metabolites were clustered based on Euclidean distances in abundance profiles across all 30 genotypes (Interactive heatmaps and associated data sets can be accessed via https://ascm.shinyapps. io/BAS_ gobrachy/).

b

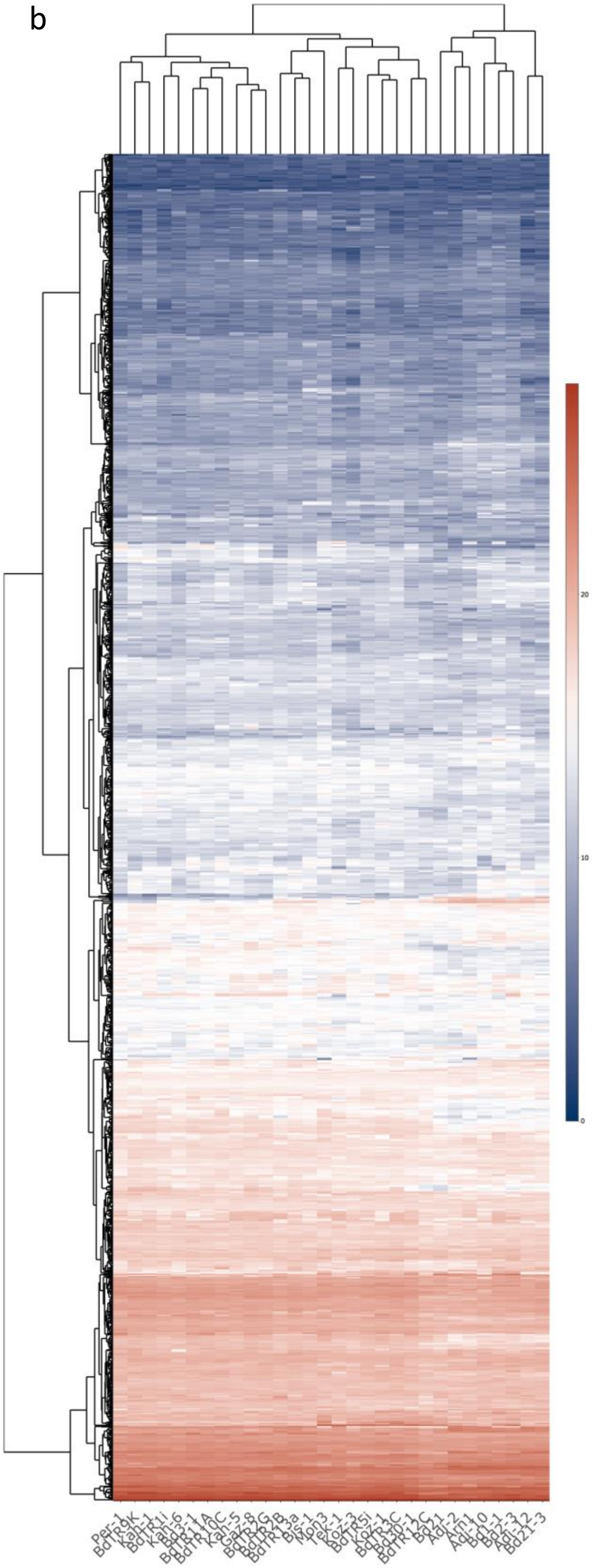

Supplementary Data Figure 5b. Metabolomics diversity of the 30 Brachypodium genotypes. The average log 2 abundances for each metabolite and accession exposed to the control treatment are plotted in two heatmaps, one for each tissue, aboveground (A) and belowground (B) biomass, where the colors indicate the abundance of each metabolite. The metabolites and genotypes were clustered hierarchically using Ward's method. Genotypes were clustered based on the Euclidean distance between metabolite profiles in the respective groups and metabolites were clustered based on Euclidean distances in abundance profiles across all 30 genotypes (Interactive heatmaps and associated data sets can be accessed via https://ascm. shinyapps.io/ BAS_gobrachy/).

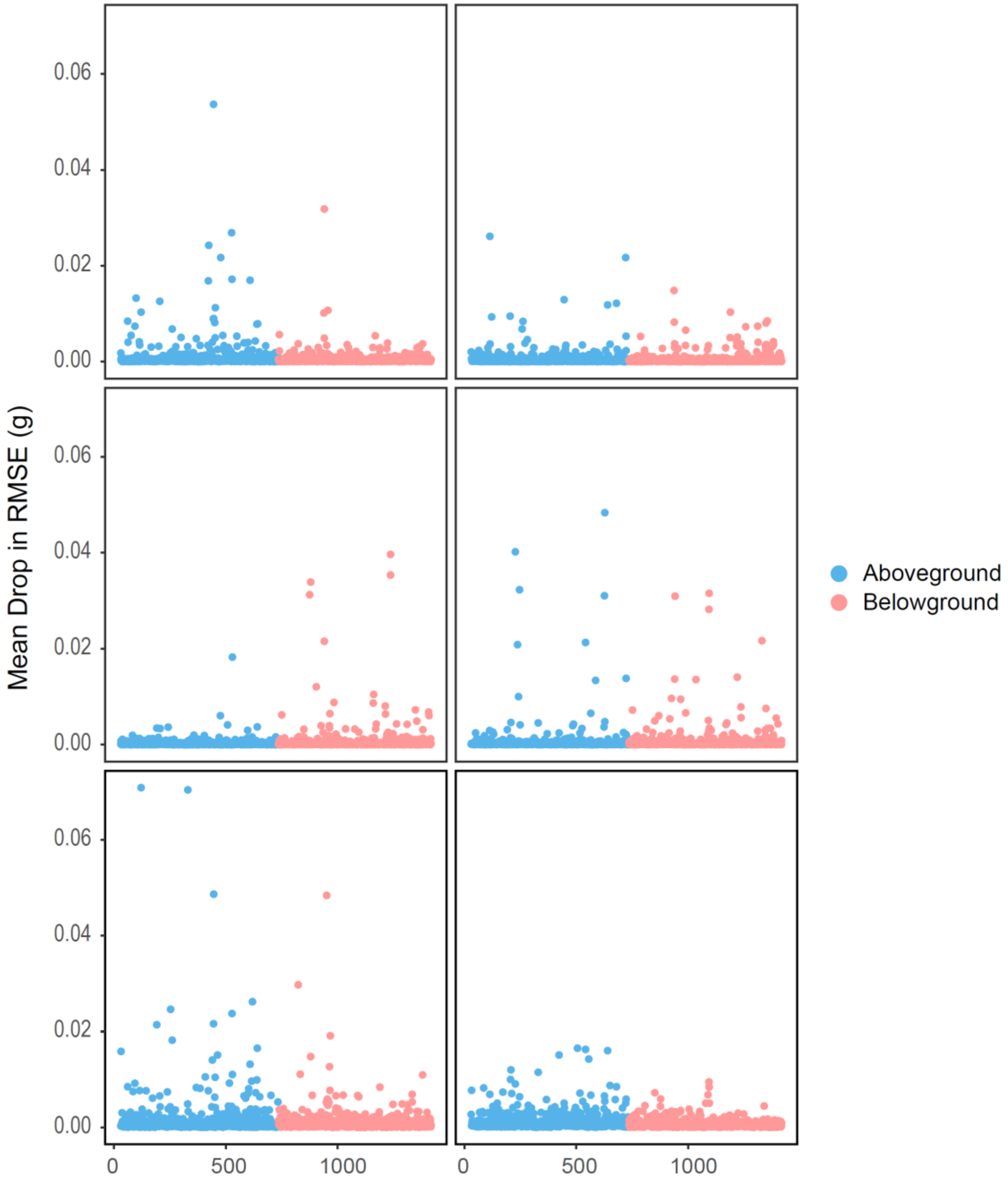

Supplementary Data Fig. 6. Variable important scores for metabolites with increased significance for aboveground, belowground and total biomass changes under control and drought conditions. Contributions by aboveground and belowground biomass are indicated by blue and pink dots, respectively.

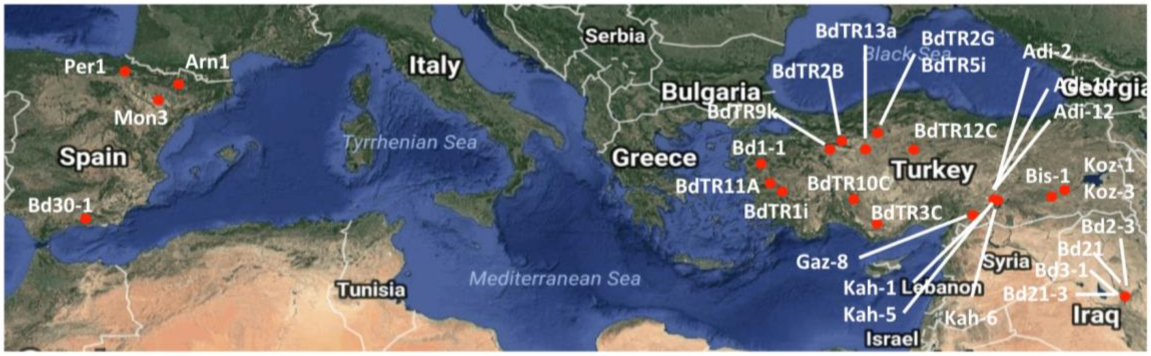

Supplementary Data Fig. 7. Map of geographic distribution for Brachypodium accessions included in this study. This map was created using version 2.6.1 of the R package ggmap. (URL: https://github.com/dkahle/ggmap); Kahle, D. and Wickham, H. (2013). ggmap: Spatial Visualization with ggplot2. The R Journal, 5(1), 144-161. Map data: copyright Google 2017; Imagery: copyright TerraMetric 2017.

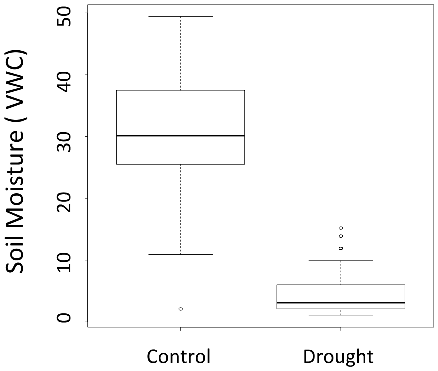

Extended Data Fig. 8. Average soil moisture of control and drought treatments. Soil moisture was recorded as the volumetric water content (VWC). The boxes show the interquartile range, the whiskers show the full range excluding outliers, and the black horizontal line represents the median of each distribution. Open circles represent outliers. n = 300.

Metabotyping as a Stopover in Genome-to-Phenome Mapping

Pubudu, P. Handakumbura[1], Bryan Stanfill[2], Albert Rivas-Ubach[1], Dan Fortin[2], John Vogel[3], Christer Jansson[1]

Supplementary Data Table 1. Average dry weight (dw) of thirty Brachypodium genotypes under control and drought conditions. Average of five biological replicates are provided. Standard deviation of each average is given in parenthesis next to each average value. Differences in dw biomass highlighted in red color are statistically significant at 95% confidence level.

	Aboveground dw (g)			Belowground dw (g)			Total dw (g)		
Accession	Control	Drought	Difference	Control	Drought	Difference	Control	Drought	Difference
Mon3	0.177 (0.022)	0.134 (0.018)	**0.043**	0.221 (0.013)	0.492 (0.046)	**-0.2714**	0.397 (0.027)	0.626 (0.037)	**-0.2284**
Adi-2	0.16 (0.057)	0.184 (0.03)	-0.024	0.136 (0.029)	0.234 (0.054)	**-0.098**	0.296 (0.05)	0.418 (0.083)	-0.122
Bd1-1	0.186 (0.055)	0.246 (0.039)	-0.0596	0.069 (0.041)	0.126 (0.015)	-0.057	0.255 (0.09)	0.371 (0.052)	-0.1166
Tek-1	0.18 (0.022)	0.222 (0.043)	-0.04135	0.09 (0.014)	0.132 (0.009)	**-0.0415**	0.271 (0.035)	0.354 (0.048)	-0.08285
Arn1	0.101 (0.013)	0.109 (0.023)	-0.0082	0.141 (0.02)	0.211 (0.043)	**-0.0696**	0.242 (0.017)	0.32 (0.056)	-0.0778
BdTR2B	0.237 (0.097)	0.238 (0.045)	-0.0016	0.09 (0.034)	0.163 (0.025)	**-0.073**	0.327 (0.125)	0.402 (0.064)	-0.0746
Kah-5	0.188 (0.085)	0.204 (0.013)	-0.0158	0.084 (0.016)	0.129 (0.024)	**-0.0446**	0.272 (0.093)	0.332 (0.029)	-0.0604
Adi-10	0.236 (0.066)	0.228 (0.044)	0.008	0.054 (0.015)	0.11 (0.012)	**-0.056**	0.29 (0.078)	0.338 (0.053)	-0.048
Bd21-3	0.331 (0.023)	0.345 (0.089)	-0.0136	0.089 (0.013)	0.121 (0.031)	-0.0312	0.421 (0.034)	0.465 (0.11)	-0.0448
BdTR13a	0.185 (0.041)	0.199 (0.031)	-0.0134	0.079 (0.01)	0.093 (0.014)	-0.0146	0.264 (0.049)	0.292 (0.039)	-0.028
Bd2-3	0.307 (0.069)	0.27 (0.063)	0.037	0.124 (0.034)	0.147 (0.037)	-0.0222	0.432 (0.094)	0.417 (0.091)	0.0148
Koz-3	0.183 (0.073)	0.181 (0.015)	0.0018	0.116 (0.03)	0.098 (0.009)	0.0182	0.299 (0.089)	0.279 (0.018)	0.02
Kah-1	0.202 (0.026)	0.191 (0.009)	0.0102	0.091 (0.005)	0.098 (0.006)	-0.0074	0.292 (0.03)	0.27 (0.037)	0.0224
BdTR12C	0.194 (0.059)	0.162 (0.046)	0.0322	0.206 (0.056)	0.212 (0.036)	-0.0066	0.399 (0.09)	0.374 (0.052)	0.0256
Bis-1	0.258 (0.064)	0.196 (0.05)	0.062	0.109 (0.02)	0.141 (0.02)	-0.0314	0.367 (0.077)	0.337 (0.066)	0.0306
BdTR9K	0.338 (0.077)	0.266 (0.044)	0.0722	0.119 (0.015)	0.16 (0.018)	**-0.0406**	0.457 (0.084)	0.426 (0.058)	0.0316
Bd3-1	0.379 (0.039)	0.327 (0.118)	0.0522	0.119 (0.009)	0.131 (0.005)	-0.0114	0.498 (0.038)	0.458 (0.121)	0.0408

BdTR5i	0.266 (0.066)	0.19 (0.027)	0.0756	0.244 (0.088)	0.274 (0.034)	-0.0308	0.509 (0.132)	0.464 (0.041)	0.0448
Kah-6	0.191 (0.022)	0.153 (0.039)	0.0376	0.102 (0.029)	0.092 (0.019)	0.0106	0.293 (0.043)	0.245 (0.049)	0.0482
BdTR3C	0.32 (0.043)	0.237 (0.054)	0.0826	0.133 (0.015)	0.162 (0.023)	-0.0288	0.453 (0.046)	0.399 (0.067)	0.0538
BdTR2G	0.198 (0.009)	0.146 (0.029)	**0.0526**	0.196 (0.04)	0.19 (0.039)	0.0062	0.394 (0.048)	0.335 (0.051)	0.0588
Koz-1	0.295 (0.045)	0.201 (0.037)	**0.094**	0.188 (0.021)	0.219 (0.016)	-0.031	0.483 (0.053)	0.42 (0.03)	0.063
BdTR11A	0.252 (0.068)	0.175 (0.04)	0.0768	0.124 (0.041)	0.136 (0.017)	-0.0122	0.376 (0.103)	0.311 (0.056)	0.0646
BdTR10C	0.275 (0.054)	0.219 (0.052)	0.0552	0.14 (0.02)	0.129 (0.016)	0.0112	0.414 (0.059)	0.348 (0.066)	0.0664
Gaz-8	0.165 (0.031)	0.116 (0.024)	0.0494	0.161 (0.029)	0.14 (0.03)	0.021	0.327 (0.039)	0.256 (0.047)	0.0704
Adi-12	0.328 (0.108)	0.228 (0.063)	0.1	0.134 (0.052)	0.116 (0.059)	0.018	0.462 (0.125)	0.344 (0.105)	0.118
Bd30-1	0.219 (0.073)	0.151 (0.077)	0.068	0.28 (0.123)	0.226 (0.092)	0.0538	0.499 (0.192)	0.377 (0.169)	0.1218
Per-1	0.207 (0.021)	0.145 (0.039)	0.0624	0.25 (0.011)	0.189 (0.036)	**0.0614**	0.457 (0.022)	0.333 (0.067)	**0.1238**
BdTR1i	0.273 (0.06)	0.144 (0.067)	**0.129**	0.107 (0.025)	0.101 (0.036)	0.0058	0.379 (0.073)	0.244 (0.06)	**0.1348**
Bd21	0.398 (0.116)	0.207 (0.082)	0.1906	0.214 (0.027)	0.222 (0.031)	-0.008	0.612 (0.13)	0.429 (0.076)	0.1826

Supplementary Data Table 2. Full factorial PERMANOVA of the entire metabolomics dataset including all 2,897 metabolite features and factors with their interactions: Water regime; Genotype; Tissue type; Water regime × Genotype; Water regime × Tissue type; Genotype × Tissue type; Water regime × Genotype × Tissue type.

	Degrees of Freedom	Sums of Squares	Mean Square	Psuedo-F	*P*
Water regime	1	$1.21{\times}10^{18}$	$1.21{\times}10^{18}$	42.23	< 0.0001
Genotype	29	$7.97{\times}10^{18}$	$2.75{\times}10^{17}$	9.58	< 0.0001
Tissue type	1	$1.12{\times}10^{19}$	$1.12{\times}10^{19}$	389.98	< 0.0001
Water regime × Genotype	29	$3.06{\times}10^{18}$	$1.06{\times}10^{17}$	3.68	< 0.0001
Water regime × Tissue type	1	$3.94{\times}10^{17}$	$3.94{\times}10^{17}$	13.73	< 0.0001
Genotype × Tissue type	29	$5.86{\times}10^{18}$	$2.02{\times}10^{17}$	7.04	< 0.0001
Water regime × Genotype × Tissue type	29	$2.89{\times}10^{18}$	$9.98{\times}10^{16}$	3.48	< 0.0001
Residuals	480	$1.38{\times}10^{19}$	$2.87{\times}10^{16}$		
Total	599	$4.64{\times}10^{19}$			

Supplementary Data Table 3. Differential abundance of aboveground metabolites in drought vs control. (Provided as a link to a .csv via https://ascm.shinyapps.io/BAS_gobrachy/).

Supplementary Data Table 4. Differential abundance of belowground metabolites in drought vs control. (Provided as a link to a .csv via https://ascm.shinyapps.io/BAS_gobrachy/).

Supplementary Data Table 5. Counts of the number of clusters corresponding to each of the 30 Brachypodium accessions based on heirachical clusterting of metabolite profiles. Thirty clusters were created using hierarchical clustering of metabolites measured by GC- and LC-MS for aboveground and belowground biomass for each plant. For each accession the table shows the number of clusters containing that accession for the control group, the drought group, and the difference between these values.

Genotype	Control	Drought	Difference
Tek-1	1	1	0
Per-1	1	1	0
Mon3	1	2	-1
Koz-3	2	2	0
Koz-1	1	1	0
Kah-6	3	2	1
Kah-5	2	1	1
Kah-1	1	1	0
Gaz-8	3	1	2
Bis-1	3	2	1
BdTR9K	1	1	0
BdTR5i	1	2	-1
BdTR3C	2	2	0
BdTR2G	2	2	0
BdTR2B	2	2	0
BdTR1i	1	3	-2
BdTR13a	3	2	1

BdTR12C	1	1	0
BdTR11A	2	2	0
BdTR10C	1	2	-1
Bd30-1	2	1	1
Bd3-1	1	3	-2
Bd21-3	2	4	-2
Bd21	1	2	-1
Bd2-3	2	1	1
Bd1-1	1	1	0
Arn1	1	1	0
Adi-2	1	2	-1
Adi-12	1	1	0
Adi-10	1	2	-1
average	1.57	1.7	-0.13

Supplementary Data Table 6. RF model predictions.

**Biomass**	**Metabolites Used**	**Control**	**Drought**
Total	All	*R2* = 0.5443739<br><br>RMSE = 0.08695723	*R2* = 0.3476867<br><br>RMSE = 0.08625607
	Belowground only	*R2* = 0.4137749<br><br>RMSE = 0.09402334	*R2* = 0.2773617<br><br>RMSE = 0.08918945
	Aboveground only	*R2* = 0.513945<br><br>RMSE = 0.08656078	*R2* = 0.3597957<br><br>RMSE = 0.08530014
	Accessions only	*R2* = 0.4144887<br><br>RMSE = 0.09139521	*R2* = 0.406699<br><br>RMSE = 0.07850669
Aboveground	All	*R2* = 0.5275662<br><br>RMSE = 0.06510273	*R2* = 0.4854229<br><br>RMSE = 0.05507526
	Belowground only	*R2* = 0.3933708<br><br>RMSE = 0.07183783	*R2* = 0.3464272<br><br>RMSE = 0.05951427
	Aboveground only	*R2* = 0.4879063<br><br>RMSE = 0.06589693	*R2* = 0.3614759<br><br>RMSE = 0.05843697
	Accessions only	*R2* = 0.4595737<br><br>RMSE = 0.06534592	*R2* = 0.4351932<br><br>RMSE = 0.055228
Belowground	All	*R2* = 0.6475371<br><br>RMSE = 0.04324062	*R2* = 0.6096695<br><br>RMSE = 0.05552345
	Belowground only	*R2* = 0.6333892<br><br>RMSE = 0.0431026	*R2* = 0.6232609<br><br>RMSE = 0.05357684
	Aboveground only	*R2* = 0.4451637<br><br>RMSE = 0.05289013	*R2* = 0.5087983<br><br>RMSE = 0.05848664

	Accessions only	*R2* =0.6176295<br>RMSE =0.04389715	*R2* = 0.7803674<br>RMSE = 0.0380778

Supplementary Data Table 7. The top-twenty important metabolites for RF model biomass predictions. Identified metabolites are shown in bold. AGBM, aboveground biomass; BGBM, belowground biomass; Unk, unknown metabolite.

Rank	AGBM-Control	AGBM-Drought	BGBMs-Control	BGBM-Drought	TBM-Control	TBM-Drought
1	L-Unk-675	**L-shikimic-acid**	R-Unk-958	L-Unk-1056	**L-tryptophan**	L-Unk-769
2	R-Unk-263	L-Unk-1822	R-Unk-957	L-Unk-209	L-Unk-425	L-Unk-854
3	L-Unk-806	R-Unk-260	R-Unk-132	L-Unk-250	L-Unk-676	L-Unk-1162
4	L-Unk-635	L-Unk-675	R-Unk-124	R-Unk-676	R-Unk-297	L-Unk-634
5	L-Unk-727	L-Unk-1382	R-Unk-264	L-Unk-1053	**R-trehalose**	L-Unk-888
6	L-Unk-808	L-Unk-1162	L-Unk-810	R-Unk-264	L-Unk-1042	L-Unk-177
7	L-Unk-1010	R-Unk-882	R-Unk-190	R-Unk-675	L-Unk-262	L-Unk-425
8	L-Unk-631	L-Unk-171	R-Unk-792	R-Unk-1283	L-Unk-808	L-Unk-175
9	**L-phenylacetic acid**	**L-tryptophan**	R-Unk-381	L-Unk-854	L-Unk-675	R-Unk-675
10	L-Unk-171	R-Unk-1376	R-Unk-791	L-Unk-229	L-Unk-134	L-Unk-209
11	L-Unk-687	L-Unk-285	R-Unk-922	R-Unk-932	R-Unk-342	L-Unk-1250
12	R-Unk-305	R-Unk-261	R-Unk-1366	L-Unk-1823	L-Unk-284	L-Unk-1382
13	**L-tryptophan**	R-Unk-1366	R-Unk-1782	R-Unk-263	L-Unk-1173	R-Unk-676
14	R-Unk-262	R-Unk-1203	R-Unk-341	R-Unk-504	**L-2,3-dihydroxybenzoate**	**L-maleic-acid**
15	L-Unk-676	R-Unk-1013	R-Unk-923	L-Unk-944	L-Unk-704	**L-2,3-dihydroxybenzoate**
16	L-Unk-673	L-Unk-282	**R-asparagine**	L-Unk-236	R-Unk-132	L-Unk-96
17	**L-galactose**	R-Unk-387	L-Unk-725	R-Unk-230	L-Unk-667	R-Unk-60
18	L-Unk-685	L-Unk-1826	R-Unk-1786	R-Unk-341	L-Unk-1010	L-Unk-784
19	L-Unk-1181	**R-leucine**	R-Unk-1376	R-Unk-957	R-Unk-336	L-Unk-184
20	L-Unk-1154	R-Unk-932	R-Unk-1033	R-Unk-1366	R-Unk-17	**L-shikimic acid**

Supplementary Data Table 8. Details of Brachypodium accessions used in this study. *Coordinate not available.

Line No	Accession	Collection location	Country	Latitude	Longitude	Drought tolerance/sensitivity according to Luo et al., 2014	Included in re-sequncing	References for each accession
1	Adi-2	Adiyaman	Turkey	37° 46' 14.5"N	38° 21' 8.2"E	M	Y	Luo et al., 2014
2	Adi-10	Adiyaman	Turkey	37° 46' 14.5"N	38° 21' 8.2"E	T	Y	Luo et al., 2011; Tyler et al., 2014; Chochois et al., 2015; Fisher et al., 2016
3	Adi-12	Adiyaman	Turkey	37° 46' 14.5"N	38° 21' 8.2"E		Y	Tyler et al., 2014; Chochois et al., 2015; Fisher et al., 2016
4	Bd21	near Salakudin	Iraq	33° 45' 39.18"	44° 24' 11.07"	S	Y	Luo et al., 2011; Tyler et al., 2014; Tyler et al., 2016; Gordon et al., 2014; Ream et al., 2014; Garvin et al., 2008; Vogel et al., 2006; Chochois et al., 2015; Fisher et al., 2016
5	Bd1-1	Turkey	Turkey	39° 11' 27.44"	27° 36' 28.59"	T	Y	Luo et al., 2011; Tyler et al., 2014; Tyler et al., 2016; Gordon et al., 2014; Ream et al., 2014; Garvin et al., 2008; Vogel et al., 2006; Chochois et al., 2015
6	Bd2-3	Iraq	Iraq	33° 45' 39.18"	44° 24' 11.07"	MS	Y	Luo et al., 2011; Tyler et al., 2014; Ream et al., 2014; Garvin et al., 2008; Vogel et al., 2006; Fisher et al., 2016
7	Arn1	Arens, Huesca	Spain	n42.25651	e0.72985		Y	Chochois et al., 2015

8	Bd21-3	near Salakudin	Iraq	33° 45' 39.18"	44° 24' 11.07"	M	Y	Luo et al., 2011; Tyler et al., 2014; Tyler et al., 2016; Gordon et al., 2014; Ream et al., 2014; Vogel et al., 2008; Vogel and Hill, 2008; Chochois et al., 2015; Fisher et al., 2016
9	Bd3-1	Iraq	Iraq	33° 45' 39.18"	44° 24' 11.07"	MS	Y	Luo et al., 2011; Tyler et al., 2014; Tyler et al., 2016; Gordon et al., 2014; Ream et al., 2014; Garvin et al., 2008; Chochois et al., 2015
10	Bd30-1	Spain	Spain	36° 59' 25.76"N	3° 33' 31.44"W	S	Y	Luo et al., 2011; Tyler et al., 2016; Gordon et al., 2014; Schwartz et al., 2010; Chochois et al., 2015
11	BdTR10C	Turkey	Turkey	37° 46'41.64"N	31° 53'5.68"E	S	Y	Luo et al., 2011; Tyler et al., 2014; Chochois et al., 2015
12	BdTR11A	Turkey	Turkey	38° 25'0.42"N	28° 1'52.75"E	M	Y	Chochois et al., 2015
13	BdTR12C	Turkey	Turkey	39° 44'53.45"N	34° 39'1.15"E	S	Y	Luo et al., 2011; Tyler et al., 2014; Gordon et al., 2014; Ream et al., 2014; Chochois et al., 2015
14	BdTR13a	Turkey	Turkey	39° 45'23.35"N	32° 25'56.46"E	M	Y	Luo et al., 2011; Tyler et al., 2014
15	BdTR2B	Turkey	Turkey	40° 4'55.55"N	31° 19'52.01"E	MS	Y	Luo et al., 2011; Chochois et al., 2015
16	BdTR2G	Turkey	Turkey	40° 23'37.13"N	32° 59'7.32"E		Y	Tyler et al., 2014; Chochois et al., 2015
17	BdTR1i	Turkey	Turkey	38° 5'35.03"N	28° 34'59.02"E		Y	Tyler et al., 2014
18	BdTR3C	Turkey	Turkey	36° 46'58.92"N	32° 57'46.71"E		Y	Tyler et al., 2014; Chochois et al., 2015

19	BdTR5i	Turkey	Turkey	40° 23'37.13"N	32° 59'7.32"E		Y	Tyler et al., 2014
20	BdTR9K	Turkey	Turkey	39° 45'10.62"N	30° 47'19.07"E	S	Y	Luo et al., 2011; Tyler et al., 2014; Chochois et al., 2015
21	Bis-1	Bismil	Turkey	37° 52' 35.6"N	41° 0' 54.3"E	S	Y	Luo et al., 2011; Chochois et al., 2015, Fisher et al., 2016
22	Gaz-8	Gaziantep	Turkey	37° 7' 39.8"N	37° 23' 26.9"E	S	Y	Luo et al., 2011; Tyler et al., 2014; Fisher et al., 2016
23	Kah-1	Kahta	Turkey	37° 44' 2.3"N	38° 32' 0.2"E	S	Y	Luo et al., 2011; Chochois et al., 2015; Fisher et al., 2016
24	Kah-5	Kahta	Turkey	37° 44' 2.3"N	38° 32' 0.2"E		Y	Tyler et al., 2014; Fisher et al., 2016
25	Koz-1	Kozluk	Turkey	38° 9' 8.2.6"N	41° 36' 34.8"E	S	Y	Luo et al., 2011; Tyler et al., 2014; Chochois et al., 2015; Fisher et al., 2016
26	Koz-3	Kozluk	Turkey	38° 9' 8.2.6"N	41° 36' 34.8"E		Y	Tyler et al., 2014; Gordon et al., 2014; Ream et al., 2014; Garvin et al., 2009; Chochois et al., 2015; Fisher et al., 2016
27	Mon3	Puetro de Pallaruelo, Castejon de Monegros. Zaragoza	Spain	N41.65132	W0.21042		Y	Fisher et al., 2016
28	Per-1	Puerto de Perdom, Navarra	Spain	N42.73704	W1.74961		Y	Fisher et al., 2016
29	Kah-6		Turkey	37° 44' 2.3"N	38° 32' 0.2"E	S	Y	Luo et al., 2011; Tyler et al., 2014
30	Tek-1*		Turkey					Tyler et al., 2014; Chochois et al., 2015

Chochois V, Vogel JP, Rebetzke GJ, Watt M. 2015. Variation in Adult Plant Phenotypes and Partitioning among Seed and Stem-Borne Roots across Brachypodium distachyon Accessions to Exploit in Breeding Cereals for Well-Watered and Drought Environments. *Plant Physiol.* 168:953-67

Fisher LHC, Han JW, Corke FMK, Akinyemi A, Didion T, et al. 2016. Linking Dynamic Phenotyping with Metabolite Analysis to Study Natural Variation in Drought Responses of Brachypodium distachyon. *Front Plant Sci* 7

Garvin DF, Gu YQ, Hasterok R, Hazen SP, Jenkins G, et al. 2008. Development of genetic and genomic research resources for Brachypodium distachyon, a new model system for grass crop research. *Crop Sci.* 48:S69-S84

Gordon SP, Priest H, Marais DLD, Schackwitz W, Figueroa M, et al. 2014. Genome diversity in Brachypodium distachyon: deep sequencing of highly diverse inbred lines. *Plant J.* 79:361-74

Luo N, Liu JX, Yu XQ, Jiang YW. 2011. Natural variation of drought response in Brachypodium distachyon. *Physiol. Plant.* 141:19-29

Ream TS, Woods DP, Schwartz CJ, Sanabria CP, Mahoy JA, et al. 2014. Interaction of Photoperiod and Vernalization Determines Flowering Time of Brachypodium distachyon. *Plant Physiol.* 164:694-709

Schwartz CJ, Doyle MR, Manzaneda AJ, Rey PJ, Mitchell-Olds T, Amasino RM. 2010. Natural Variation of Flowering Time and Vernalization Responsiveness in Brachypodium distachyon. *Bioenergy Research* 3:38-46

Tyler L, Fangel JU, Fagerstrom AD, Steinwand MA, Raab TK, et al. 2014. Selection and phenotypic characterization of a core collection of Brachypodium distachyon inbred lines. *BMC Plant Biol.* 14

Tyler L, Lee SJ, Young ND, DeIulio GA, Benavente E, et al. 2016. Population Structure in the Model Grass *Brachypodium distachyon* Is Highly Correlated with Flowering Differences across Broad Geographic Areas. *Plant Genome-Us* 9

Vogel J, Hill T. 2008. High-efficiency Agrobacterium-mediated transformation of Brachypodium distachyon inbred line Bd21-3. *Plant Cell Rep.* 27:471-8

Vogel JP, Gu YQ, Twigg P, Lazo GR, Laudencia-Chingcuanco D, et al. 2006. EST sequencing and phylogenetic analysis of the model grass Brachypodium distachyon. *Theor. Appl. Genet.* 113:186-95

Vogel JP, Tuna M, Budak H, Huo NX, Gu YQ, Steinwand MA. 2009. Development of SSR markers and analysis of diversity in Turkish populations of Brachypodium distachyon. *BMC Plant Biol.* 9

Supplementary Data Table 9. Parameters applied to GC-MS chromatograms with Metabolite Detector 2.5 for metabolomic profiling.

**Tool settings**		
Centroid	Threshold begin	10
	Peak threshold end	-5
	Maximal baseline	30
	FWHM	0.1
Deconvolution	Peak threshold	10
	Minimum peak height	10
	Deconvolution width (scans)	8
Identification	Max RI difference	20
	Cutoff score	0.6
	Pure/Impure	0.6
	Scaled lib	Yes
	Combined score	Yes
Quantification	Minimal distance	0.5
	Minimal required quality index	1
	Exclude	72.5 to 73.5<br>146.5 to 147.5
**Batch quantification Settings**		
Compound matching	ARI	20
	Pure/Impure	0.6
	Req. Score	0.8
	RI+Spec	OK
Identification	ARI	20
	Pure/Impure	0.6
	RI+Spec	OK
Other settings	Compound reproducibility	0
	Max. Peak drisc. index	100
	S/N	15
	Number of ions	4
	Extended SIC Scan	Yes

Supplementary Data Table 10. Score, average retention index (RI), average retention time (RT) and average signal to noise ratio (S/N) of matched metabolites in GC-MS chromatograms processed with Metabolite Detector 2.5 for metabolite matches verified and non-verified by NIST compound library.

Metabolite matches verified with NIST library and included in the study						Metabolite matches not verified with NIST library and thus, not included in the study				
Metabolite name in Database	Metabolite Detector Score	Average RI	Average RT (Min)	Average S/N		Metabolite	Metabolite Detector Score	Average RI	Average RT (Min)	Average S/N
fumaric acid	0.99	1024.1	10.94	272.6		palatinose	0.86	2515.3	25.39	28.3
citric acid	0.97	1518.8	16.88	293.7		2-hydroxypyridine	0.85	715.4	6.66	174.6
Sucrose	0.97	2376.0	24.40	520.3		urea	0.85	918.6	9.49	19.1
palmitic acid	0.97	1720.0	18.91	437.5		acetyl-L-serine 2	0.85	1079.9	11.67	21.9
phosphoric acid	0.96	954.7	10.00	167.6		adenine 1	0.85	1556.3	17.28	72.8
galactonic acid	0.96	1709.2	18.81	196.3		gentisic acid	0.85	1457.5	16.22	29.9
D-glucose 1	0.96	1611.1	17.86	189.2		xylitol	0.85	1418.8	15.80	7.1
D-glucose 2	0.95	1630.0	18.04	77.9		L-asparagine 2	0.84	1353.5	15.05	9.6
L-alanine 1	0.95	781.6	7.58	66.2		melibiose 2	0.84	2560.2	25.71	25.8
aspartic acid 2	0.95	1210.2	13.35	356.7		D-lyxosylamine 2	0.83	1348.6	14.99	27.0
carbonate ion	0.95	819.0	8.10	112.1		D-saccharic acid	0.83	1704.0	18.76	10.7
D-malic acid	0.95	1170.9	12.85	90.9		aspartic acid 1	0.83	1097.5	11.90	20.8
D-(+) trehalose	0.94	2486.5	25.19	357.4		porphine 1	0.83	1017.3	10.85	50.3
succinic acid	0.93	991.6	10.51	216.7		glycerol	0.83	958.7	10.05	180.2
L-mimosine 1	0.93	872.2	8.84	13.8		vanillin	0.82	1332.2	14.80	21.6
maleic acid	0.93	984.7	10.41	158.5		4-hydroxypyridine	0.82	830.3	8.26	7.3
myo-inositol	0.93	1799.1	19.68	356.1		leucrose	0.82	2463.8	25.03	42.7
campesterol	0.92	2979.9	28.48	54.5		benzoic acid	0.82	925.5	9.59	8.3
linoleic acid	0.92	1886.6	20.45	150.0		beta-glycerolphosphate	0.82	1420.4	15.82	7.6
glycine	0.92	989.6	10.48	91.8		citraconic acid 1	0.82	1032.9	11.06	9.1
ribose	0.92	1376.5	15.32	220.0		lactulose 1	0.81	2287.9	23.74	84.9
D (+) galactose 2	0.91	1637.3	18.11	142.9		L-(-)-fucose 2	0.81	1418.0	15.79	30.1
D-glucose-6-phosphate 2	0.91	2060.7	21.95	38.7		mucic acid	0.81	1714.5	18.86	44.2
stearic acid	0.91	1914.2	20.70	185.6		L-glutamic acid 1	0.81	1203.9	13.28	5.3
L-serine 2	0.91	1047.0	11.24	364.1		turanose 1	0.81	2425.8	24.76	9.1
glycolic acid	0.91	754.7	7.21	116.2		4-hydroxy-3-methoxybenzoic acid	0.81	1448.7	16.13	11.3
arachidic acid	0.9	2111.1	22.36	15.2		gluconic acid 2	0.8	1649.7	18.23	41.1
L-(+) lactic acid	0.9	741.6	7.02	185.5		D-allose 1	0.79	1553.2	17.25	9.3
L-proline 2	0.9	980.7	10.36	214.1		L-ornithine 1	0.79	1301.8	14.44	37.0
glyceric acid	0.9	1014.1	10.81	173.3		sarcosine	0.78	815.4	8.05	22.8
L-threonine 2	0.9	1075.3	11.61	298.4		methyl vanillate	0.78	1337.5	14.86	6.0
Phenylalanine 2	0.9	1317.1	14.62	182.3		salicylic acid	0.78	1201.0	13.24	6.2

maltose 1	0.9	2407.0	24.63	60.6		caprylic acid	0.78	938.9	9.78	12.2
1-aminocyclopropane-1-carboxylic acid	0.9	883.0	9.00	41.0		melibiose 1	0.77	2530.7	25.50	101.9
D-mannitol	0.9	1641.9	18.16	27.9		arbutin	0.77	2243.2	23.41	12.1
L-glutamic acid 2	0.89	1309.2	14.53	430.9		L-methionine 2	0.77	1206.8	13.31	38.4
L-serine 1	0.89	936.3	9.74	69.1		phosphoenolpyruvic acid	0.76	1269.1	14.05	8.6
L-leucine 1	0.89	833.6	8.31	114.9		allantoin 3	0.76	1765.2	19.35	5.3
cellobiose 2	0.89	2472.4	25.09	40.7		palatinitol 2	0.76	2603.6	26.01	13.8
maltitol	0.89	2508.1	25.34	41.6		L-cysteine 2	0.76	1244.7	13.76	19.4
ferulic acid	0.89	1774.3	19.44	13.1		homocysteine	0.76	1352.1	15.03	7.8
iminodiacetic acid 2	0.89	1208.0	13.33	34.1		hydroquinone	0.76	1087.7	11.77	11.6
beta-cyano-L-alanine	0.88	1056.8	11.37	50.6		ribonic acid-gamma-lactone 2	0.75	1384.0	15.41	5.5
4-guanidinobutyric acid 2	0.88	1218.8	13.45	404.0		glucuronic acid 4	0.75	1602.4	17.78	21.4
L-leucine 2	0.88	954.2	9.99	90.5		carbamic acid ethyl ester (urethane)	0.75	692.6	6.34	10.5
D-fructose-6-phosphate 2	0.88	2027.4	21.68	78.8		10-hydroxydecanoic acid	0.75	1475.4	16.41	38.2
dehydroascorbic acid 2	0.88	1548.2	17.20	218.7		2-piperidone 1	0.74	809.1	7.97	229.4
lactose 2	0.88	2387.4	24.49	41.3		D-sorbitol	0.74	1613.3	17.88	23.1
uracil	0.87	1020.0	10.89	10.1		lactitol	0.74	2440.9	24.87	37.1
caffeic acid	0.87	1819.6	19.86	87.0		ethyl glucuronide	0.73	1681.0	18.54	94.6
D-ribulose-5-phosphate 2	0.87	1830.2	19.95	11.7		3-hydroxy-3-methylglutaric acid (dicrotalic acid)	0.73	1290.9	14.31	9.1
D-glucose-6-phosphate 1	0.87	2040.3	21.78	117.1		L-tyrosine 2	0.73	1635.2	18.09	76.2
pyruvic acid	0.86	730.9	6.87	124.1		phenyl-beta-glucopyranoside	0.73	1957.4	21.08	15.3
4-hydroxybenzoic acid	0.86	1311.1	14.55	13.8		tartaric acid	0.72	1298.3	14.40	24.5
L-valine 2	0.86	897.5	9.20	293.1		turanose 2	0.72	2465.7	25.04	9.3
DL-isoleucine 2	0.86	976.5	10.30	122.4		pantothenic acid 2	0.72	1687.3	18.60	7.4
3-hydroxyisobutyric acid	0.86	839.3	8.39	22.1		D-glucosaminic acid 2	0.72	1727.9	18.99	30.7
3-methoxytyramine	0.86	1729.9	19.01	68.2		3-dehydroshikimic acid 2	0.72	1479.3	16.45	10.5
ribitol	0.86	1407.1	15.68	10.2		valproic acid glucuronide	0.72	2081.6	22.12	197.6
D-xylose	0.85	1356.5	15.08	141.7		lactulose 3	0.71	2378.0	24.42	37.9
behenic acid	0.85	2308.6	23.90	16.3		2-keto-L-gulonic acid 1	0.71	1587.2	17.61	5.2
D-ribulose-5-phosphate 1	0.85	1813.0	19.80	9.7		3-aminopropionitrile 2	0.71	948.4	9.91	242.6
L-glutamic acid 3 (dehydrated)	0.84	1212.6	13.38	238.4		galactinol 1	0.71	2609.4	26.05	64.2
trans-aconitic acid	0.83	1432.1	15.95	31.8		O-phosphocolamine	0.7	1479.0	16.45	41.0
cellobiose 1	0.82	2447.0	24.91	158.6		ribonic acid-gamma-lactone 1	0.7	1361.6	15.15	164.5
L-pyroglutamic acid	0.82	1200.2	13.23	45.0		L-methionine sulfoxide 2	0.7	1468.9	16.34	85.3
L-proline 2	0.81	978.3	10.33	40.4		D-sphingosine 3	0.7	2119.9	22.43	17.3
glycerol 1-phosphate	0.81	1459.9	16.25	243.3		chlorogenic acid 2	0.7	2772.6	27.12	14.1

shikimic acid	0.81	1501.2	16.69	281.8		N-acetyl-D-mannosamine 1	0.7	1733.3	19.04	283.4
nicotinic acid	0.81	973.1	10.25	20.2		galacturonic acid 2	0.69	1654.6	18.28	7.9
L-homoserine 2	0.8	1136.9	12.41	17.0		L-(-)-fucose 1	0.68	1417.7	15.79	5.9
alpha ketoglutaric acid	0.8	1263.0	13.98	30.7		D-sphingosine 2	0.66	2112.9	22.37	26.7
L-proline 1	0.8	850.8	8.55	12.4		3-hydroxypropanoic acid 1	0.66	1253.5	13.86	5.3
L-glutamine 2	0.8	1286.5	14.26	103.2		2-hydroxybiphenyl	0.66	1287.8	14.27	9.9
O-acetylsalicylic acid	0.79	1197.8	13.20	19.9		DL-glyceraldehyde 3-phosphate 2	0.66	1391.7	15.50	10.7
L-glutamine 3	0.78	1465.1	16.30	77.1		spermidine 2	0.65	1948.8	21.00	11.0
D-lyxose 2	0.77	1347.4	14.98	20.0		glucuronic acid 3	0.65	1586.9	17.61	13.2
L-asparagine 1	0.76	1323.9	14.70	197.9		asp-glu 2	0.65	1895.6	20.53	229.9
phytol 2	0.76	1850.5	20.13	129.9		L-mimosine 2	0.65	1124.0	12.24	58.4
L-tryptophan 2	0.76	1917.9	20.73	214.8		tartronic acid	0.65	1084.6	11.73	15.4
quinic acid	0.73	1565.5	17.38	413.7		alpha-D-glucosamine 1-phosphate	0.65	1507.9	16.76	27.6
dopamine (hydroxytyramine)	0.72	1774.6	19.44	482.4		O-phospho-L-serine 2	0.64	1508.6	16.77	14.6
N-acetyl-D-mannosamine 2	0.71	1788.1	19.57	50.3		dehydroascorbic acid 6	0.63	1640.5	18.14	8.1
L-valine 1	0.69	765.7	7.36	52.2		N-acetyl-D-glucosamine 2	0.62	1788.6	19.58	6.9
serotonin 2	0.68	2157.5	22.74	348.0		O-phospho-L-threonine 1	0.62	1448.6	16.12	16.9
L-ascorbic acid	0.66	1650.1	18.24	23.0		8-aminocaprylic acid	0.62	1601.8	17.77	88.2
						4-methyl-5-thiazoleethanol	0.61	1036.5	11.10	14.7
						2-deoxyuridine 2	0.61	2102.7	22.29	11.9
						N-gamma-acetyl-N-2-formyl-5-methoxykynurenamine 3	0.61	2022.5	21.64	174.7
						4-acetamidobutyric acid 1	0.61	1162.6	12.74	12.2
						cytidine 2	0.61	2474.8	25.11	80.4
						alpha-glucosamine 1-phosphate	0.61	1480.2	16.46	9.2
						malonamide 1	0.61	1084.0	11.72	18.2
						DL-dihydrosphingosine 3	0.6	2195.9	23.05	5.2

Supplementary Data Table 11. LC-MS chromatogram processing by MZmine 2.26. The table summarizes the different parameters and processes to the LC-MS chromatogram files.

			(-H) Chromatograms
1	**Baseline correction**		
	Chromatogram type		TIC
	MS level		1
	Correction method		RollingBall baseline corrector<br>vm: 10<br>ws: 12
2	**Mass detection** (exact Mass)		
	Noise level		1×10^4
3	**Chromatogram builder**		
	Minimum time span		0.08 min
	Minimum height		1000
	m/z tolerance		0.0005 Da or 7 ppm
4	**Smoothing**		
	Filter width		5
5	**Chromatogram deconvolution (local minimum search)**		
	Chromatographic threshold		40%
	Search minimum in RT range (min)		0.1
	Minimum relative height		40%
	Minimum absolute height		10000
	Minimum ratio of peak top/edge		1
	Peak duration range		0-0.5
6	**Chromatogram alignment (join alignment)**		
	m/z tolerance		0.0005 Da or 7 ppm
	Weight for m/z		100
	RT tolerance		0.25 min
	Weight for RT		60
7	**Gap filling (Peak Finder)**		
	Intensity tolerance		40%
	m/z tolerance		0.0005 Da or 7 ppm
	Retention time tolerance		0.15 min

	RT correction		Not marked
**8**	**Metabolite Assignation**		
	m/z tolerance		0.0005 Da or 7 ppm
	RT tolerance		0.25 min

RT, retention time; m/z, mass to charge ratio

Supplementary Data Table 12. Mass-to-charge ratio (m/z) and retention time (RT) of the deconvoluted ions assigned to metabolites with MZmine v.2.26 through our in-home metabolomics database. The assignment of the metabolites is based on the RT and the exact mass of standards analyzed in negative ionization mode. RT and parent ion in negative mode of the standards matching with the LC-MS chromatograms are shown. The errors for m/z (Da and ppm) and RT of the ions assigned to metabolites relative to the measurements of the standards are indicated.

	Measured deconvoluted ions			Parent ion (- mode) and RT from standards measured in the LC-MS Orbitrap			Error of m/z and RT (MZmine deconvoluted ions vs. standard measurements)		
Metabolite	m/z	RT (min)		Parent ion (m/z) Negative ionization mode	RT (min)		m/z error (Da)	m/z error (ppm)	RT error (min)
2,3-dihydroxybenzoate	153.019949	8.10		153.019334	7.84		0.000615	4.02	0.26
2,3-dihydroxybenzoate	153.019640	8.02		153.019334	7.84		0.000306	2.00	0.18
2-hydroxyphenylacetic acid	151.040405	8.87		151.040064	9		0.000341	2.26	-0.13
2-methylmaleate	129.019977	1.79		129.019334	2.05		0.000643	4.99	-0.26
3-dehydroshikimate	171.029911	1.38		171.029894	1.45		0.000017	0.10	-0.07
3-methyl-2-oxovaleric acid	129.056565	4.62		129.055724	4.62		0.000841	6.52	0.00
4-aminobutanoate; 3-aminoisobutanoate	102.056377	1.33		102.056054	1.25		0.000323	3.16	0.08
4-aminobutanoate; 3-aminoisobutanoate	102.056371	1.43		102.056054	1.25		0.000317	3.10	0.18
4-coumarate; 3-hydroxycinnamic acid	163.040273	9.13		163.040024	9.42		0.000249	1.53	-0.29
4-coumarate; 3-hydroxycinnamic acid	163.040329	9.30		163.040024	9.42		0.000305	1.87	-0.12
4-methyl-2-oxovaleric acid; 4-methyl-2-oxo-pentanoic acid	129.056537	5.67		129.055714	5.67		0.000823	6.38	0.00
5,6-dihydrouracil	113.036069	1.31		113.035654	1.36		0.000415	3.67	-0.05
5,6-dihydrouracil	113.036145	1.41		113.035654	1.36		0.000491	4.34	0.05
5,6-dihydrouracil	113.036318	1.32		113.035654	1.36		0.000664	5.87	-0.04
5-oxo-d-proline	128.035990	1.79		128.035314	1.76		0.000676	5.28	0.03
alpha-ketoglutaric acid	145.014601	1.57		145.014254	1.79		0.000347	2.39	-0.22

ascorbic acid	175.025217	1.41		175.024814	1.36		0.000403	2.30	0.05
ascorbic acid	175.024960	1.41		175.024814	1.36		0.000146	0.84	0.05
asparagine	131.046784	1.32		131.046214	1.31		0.000570	4.35	0.01
asparagine	131.047122	1.32		131.046214	1.31		0.000908	6.93	0.01
aspartate	132.030786	1.32		132.030234	1.5		0.000552	4.18	-0.18
aspartate	132.031102	1.35		132.030234	1.5		0.000868	6.57	-0.15
azelaic acid	187.098102	11.18		187.097584	11.4		0.000518	2.77	-0.22
betaine;<br>valine;<br>norvaline;<br>5-aminopentanoate	116.071952	1.52		116.071704	1.35		0.000248	2.14	0.17
citrate	191.020394	1.79		191.019734	1.78		0.000660	3.45	0.01
citrate	191.020383	1.75		191.019734	1.78		0.000649	3.40	-0.03
citrulline	174.088797	1.33		174.088414	1.32		0.000383	2.20	0.01
3-phosphoglyceric acid	184.986124	1.49		184.985659	1.64		0.000465	2.51	-0.15
glucose 6-phosphate;<br>alpha-d-glucose 1-phosphate	259.023051	1.30		259.022444	1.56		0.000607	2.34	-0.26
glucuronic acid	193.035430	1.34		193.035372	1.51		0.000058	0.30	-0.17
gulonic acid gama-lactone;<br>glucono-1,5-lactone;<br>gluconate	177.041047	1.34		177.040464	1.53		0.000583	3.29	-0.19
gulonic acid gama-lactone;<br>glucono-1,5-lactone;<br>gluconate	177.040753	1.41		177.040464	1.53		0.000289	1.63	-0.12
disaccharides (melibiose;d-(+)-<br>cellobiose;palatinose;maltose;sucrose)	341.109768	1.33		341.108944	1.24		0.000824	2.42	0.09
dopamine	152.072576	1.40		152.071703	1.35		0.000873	5.74	0.05
dopamine	152.072155	1.40		152.071703	1.35		0.000452	2.98	0.05
ornithine	131.083050	1.32		131.082598	1.26		0.000452	3.45	0.06
ferulate	193.051203	9.99		193.050634	10.28		0.000569	2.95	-0.29
fumarate	115.004196	1.39		115.003684	1.51		0.000512	4.45	-0.12
fumarate	115.004439	1.52		115.003684	1.51		0.000755	6.56	0.01
gluconic acid	195.051425	1.44		195.051024	1.52		0.000401	2.06	-0.08
gluconic acid	195.052012	1.36		195.051024	1.52		0.000988	5.06	-0.16
glutamic acid	146.046405	1.33		146.045924	1.28		0.000481	3.29	0.05
glutamic acid	146.046769	1.33		146.045924	1.28		0.000845	5.79	0.05
glutathione	306.077710	1.65		306.076534	1.53		0.001176	3.84	0.12
glyceraldehyde;<br>lactate	89.024800	1.39		89.024424	1.39		0.000376	4.22	0.00
glyceraldehyde;<br>lactate	89.024717	1.40		89.024424	1.39		0.000293	3.29	0.01
glyceric acid	105.019748	1.39		105.019334	1.58		0.000414	3.94	-0.19

glyceric acid	105.019756	1.40		105.019334	1.58		0.000422	4.02	-0.18
hesperetin	301.072952	13.43		301.071724	13.66		0.001228	4.08	-0.23
hexoses (sorbose;myo-inositol;d-galactose;mannose;alpha-d-glucose;d-sorbitol;allose;d-tagatose;d-psicose;theobromine;d-fructose)	179.056812	1.33		179.056114	1.29		0.000698	3.90	0.04
hexoses (sorbose;myo-inositol;d-galactose;mannose;alpha-d-glucose;d-sorbitol;allose;d-tagatose;d-psicose;theobromine;d-fructose)	179.056786	1.37		179.056114	1.29		0.000672	3.75	0.08
homoorientin	447.094194	9.12		447.093324	9.23		0.000870	1.95	-0.11
homoorientin	447.094022	8.99		447.093324	9.23		0.000698	1.56	-0.24
homoserine; threonine; allothreonine	118.051420	1.33		118.050964	1.31		0.000456	3.86	0.02
homoserine; threonine; allothreonine	118.051719	1.33		118.050964	1.31		0.000755	6.40	0.02
hydroxypyruvate; malonate	103.004043	1.59		103.003684	1.6		0.000359	3.49	-0.01
hydroxypyruvate; malonate	103.003809	1.78		103.003684	1.6		0.000125	1.22	0.18
hypoxanthine	135.030574	1.35		135.031234	1.38		-0.000660	-4.88	-0.03
hypoxanthine	135.030702	1.47		135.031234	1.38		-0.000532	-3.94	0.09
inosine	267.074432	1.34		267.073494	1.37		0.000938	3.51	-0.03
isocitric acid	191.020528	1.39		191.019734	1.57		0.000794	4.15	-0.18
isocitric acid	191.020586	1.48		191.019734	1.57		0.000852	4.46	-0.09
glutamine	145.062426	1.32		145.061864	1.32		0.000562	3.87	0.00
glutamine	145.062829	1.32		145.061864	1.32		0.000965	6.66	0.00
malate	133.014239	1.39		133.014254	1.54		-0.000015	-0.11	-0.15
maleamate	114.020138	1.32		114.019664	1.47		0.000474	4.15	-0.15
mannitol	181.072879	1.33		181.071764	1.3		0.001115	6.16	0.03
methylmalonate	117.019817	1.81		117.019334	2.05		0.000483	4.13	-0.24
mono-ethyl malonate;glutarate	131.035672	1.33		131.034984	1.53		0.000688	5.25	-0.20
n-acetyl-dl-glutamic acid	188.057191	1.39		188.056444	1.4		0.000747	3.97	-0.01
naringenin	271.062247	13.34		271.061224	13.33		0.001023	3.78	0.01
p-anisaldehyde; 4'-hydroxyacetophenone	135.045804	8.74		135.045124	8.89		0.000680	5.03	-0.15
p-anisaldehyde; 4'-hydroxyacetophenone	135.045446	8.63		135.045124	8.89		0.000322	2.39	-0.26
pentoses (d-xylose;d-(-)-arabinose;d-(-)-ribose;d-ribose;arabinose;d-lyxose)	149.045836	1.35		149.045554	1.32		0.000282	1.89	0.03

phenylacetic acid	135.045027	3.88		135.045154	3.82		-0.000127	-0.94	0.06
phenylacetic acid	135.044731	3.99		135.045154	3.82		-0.000423	-3.13	0.17
phloroglucinol	125.024983	1.33		125.024424	1.44		0.000559	4.47	-0.11
proline	114.056288	1.39		114.056054	1.34		0.000234	2.05	0.05
propanoate	73.029803	1.81		73.029504	1.9		0.000299	4.09	-0.09
purine	119.035743	1.33		119.036324	1.4		-0.000581	-4.88	-0.07
pyruvate	87.009062	1.75		87.008774	1.77		0.000288	3.31	-0.02
pyruvic aldehyde	71.014191	1.38		71.013854	1.38		0.000337	4.74	0.00
pyruvic aldehyde	71.014195	1.42		71.013854	1.38		0.000341	4.80	0.04
quinate	191.056432	1.43		191.056114	1.57		0.000318	1.66	-0.14
quinate	191.057088	1.42		191.056114	1.57		0.000974	5.10	-0.15
salicylate	137.024930	11.32		137.024424	11.39		0.000506	3.69	-0.07
salicylate	137.024927	11.38		137.024424	11.39		0.000503	3.67	-0.01
salicylate	137.024728	11.29		137.024424	11.39		0.000304	2.22	-0.10
serine	104.035696	1.32		104.035314	1.29		0.000382	3.67	0.03
serine	104.035966	1.32		104.035314	1.29		0.000652	6.26	0.03
shikimate	173.046104	1.40		173.045554	1.39		0.000550	3.18	0.01
glycerol 3-phosphate; glycerol 2-phosphate	171.006949	1.32		171.006394	1.61		0.000555	3.25	-0.29
succinate	117.019757	1.43		117.019334	1.38		0.000423	3.61	0.05
succinate	117.020000	1.43		117.019334	1.38		0.000666	5.69	0.05
trans-aconitate	173.009827	1.79		173.009164	1.82		0.000663	3.83	-0.03
trans-aconitate	173.009646	1.76		173.009164	1.82		0.000482	2.79	-0.06
uracil	111.019987	1.38		111.020004	1.38		-0.000017	-0.15	0.00
urocanate	137.035684	1.20		137.035654	1.33		0.000030	0.22	-0.13
urocanate	137.035361	1.34		137.035654	1.33		-0.000293	-2.14	0.01
vitexin	431.098909	9.84		431.098324	9.88		0.000585	1.36	-0.04
vitexin	431.099752	9.84		431.098324	9.88		0.001428	3.31	-0.04
